# The HVEM-BTLA Immune Checkpoint Restrains Murine Chronic Cholestatic Liver Injury by Regulating the Gut Microbiota

**DOI:** 10.3389/fimmu.2022.773341

**Published:** 2022-02-04

**Authors:** Yanbo Kou, Xingping Zheng, Liyuan Meng, Mengnan Liu, Shihong Xu, Qiyue Jing, Shenghan Zhang, Hanying Wang, Jinzhi Han, Zhuanzhuan Liu, Yanxia Wei, Yugang Wang

**Affiliations:** Laboratory of Infection and Immunity, Jiangsu Key Laboratory of Immunity and Metabolism, Department of Pathogenic Biology and Immunology, Xuzhou Medical University, Xuzhou, China

**Keywords:** bile acids, cholestasis, IgA, immune checkpoint, microbiota, neutrophils

## Abstract

The herpes virus entry mediator (HVEM) is an immune checkpoint molecule regulating immune response, but its role in tissue repair remains unclear. Here, we reported that HVEM deficiency aggravated hepatobiliary damage and compromised liver repair after 3,5-diethoxycarbonyl-1,4-dihydrocollidine (DDC)-induced injury. A similar phenotype was observed in B and T lymphocyte attenuator (BTLA)-deficient mice. These were correlated with impairment of neutrophil accumulation in the liver after injury. The hepatic neutrophil accumulation was regulated by microbial-derived secondary bile acids. HVEM-deficient mice had reduced ability to deconjugate bile acids during DDC-feeding, suggesting a gut microbiota defect. Consistently, both HVEM and BTLA deficiency had dysregulated intestinal IgA responses targeting the gut microbes. These results suggest that the HVEM-BTLA signaling may restrain liver injury by regulating the gut microbiota.

## Introduction

Cholestasis, i.e., impairment of bile formation and/or flow, is the most common clinical symptom shared by cholestatic liver diseases, such as primary biliary cirrhosis (PBC), primary sclerosing cholangitis (PSC), and secondary sclerosing cholangitis. The accumulation of hydrophobic bile acids due to bile duct obstruction is believed to trigger hepatic inflammation and injury that eventually progress to liver fibrosis and cirrhosis, a devastating end-stage condition of many chronic liver diseases (1, 2). Currently, there are only limited therapies, and the molecular and cellular mechanism by which cholestasis induces diseases remains incompletely understood. Both innate and adaptive immune cells are suggested to be involved in the pathophysiological process of cholestatic liver diseases. For example, innate immune cells such as neutrophils and macrophages can either aggravate cholestatic liver injury through producing proinflammatory cytokine and reactive oxygen species or promote liver repair by functional conversion to a reparative phenotype (3, 4). Production of interleukin 17 by intrahepatic γδ T-cell contributes to the development of hepatic fibrosis (5). It has been reported that in PBC patients there is an increase of follicular helper T cells in the blood, which is correlated with disease severity and responsiveness to ursodeoxycholic acid therapy (6). Although great progress has been made in understanding the role of the immune system during cholestasis, the relationship between immune cell reactions and cholestasis remains unclear.

The herpes virus entry mediator (HVEM), also known as CD270 or tumor necrosis factor receptor superfamily 14 (TNFRSF14), is an important immune checkpoint regulatory molecule that is expressed mainly on T cells, B cells, as well as several other cell types (7). HVEM can act as a ligand or a receptor in a cell type- and context-dependent manner to interact with multiple molecules, including two TNF superfamily members, LIGHT (lymphotoxin-like, exhibits inducible expression, and competes with HSV glycoprotein D for HVEM, a receptor expressed by T lymphocytes) and soluble Lymphotoxin (LTα3), and two immunoglobulin (Ig) superfamily members, BTLA and CD160 (8, 9). The ability of HVEM to interact with multiple partners creates a functional diversity of HVEM to engage both inflammatory and anti-inflammatory signals to regulate a variety of pathophysiological processes. Whether the HVEM networks are involved in regulating cholestatic liver diseases remains unknown.

Here, we found that the HVEM-BTLA axis acts to restrain a chemical-induced chronic cholestatic liver injury. The HVEM-BTLA axis influences mucosal IgA production and fine-tunes the gut microbiota to maintain bile acids biotransformation during cholestasis. Secondary bile acids transformed by the microbiota obtain a capacity to promote intrahepatic neutrophil accumulation, which helps to repair liver damage.

## Materials and Methods

### Animal Studies

**Figure 1:**
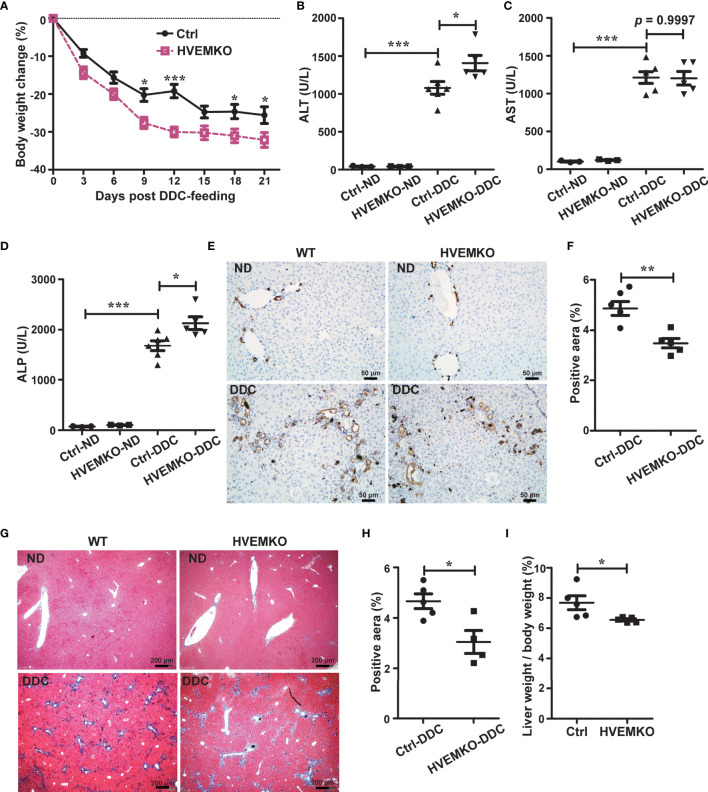
HVEM-deficiency compromises hepatic repair to DDC-induced chronic cholestatic injury. **(A)** Daily body weight changes of WT littermate controls (ctrl) and *HVEM^-/-^* mice during DDC-feeding. **(B–D)** Serum levels of ALT **(B)**, AST **(C)**, and ALP **(D)** in WT littermate controls (ctrl) and *HVEM^-/-^* mice that fed with either a normal chow diet (ND) or a DDC-containing diet for 21 days. **(E, F)** Immunohistochemistry staining of CK-19 in liver sections from the indicated mice **(E)**. The percentage of CK-19-stained positive area (brown color) per visual field was calculated accordingly **(F)**. n = 5, scale bar = 50 µm. **(G, H)** Masson’s trichrome staining for collagen fiber **(G)**. The percentage of the fibrotic area (blue color) per visual field was calculated **(H)**. n = 5, scale bar = 200 µm. **(I)** The liver-to-body weight ratios 21 days after DDC-feeding. Data were represented as the mean ± SEM. *p < 0.05; **p < 0.01; ***p < 0.001.

**Figure 2:**
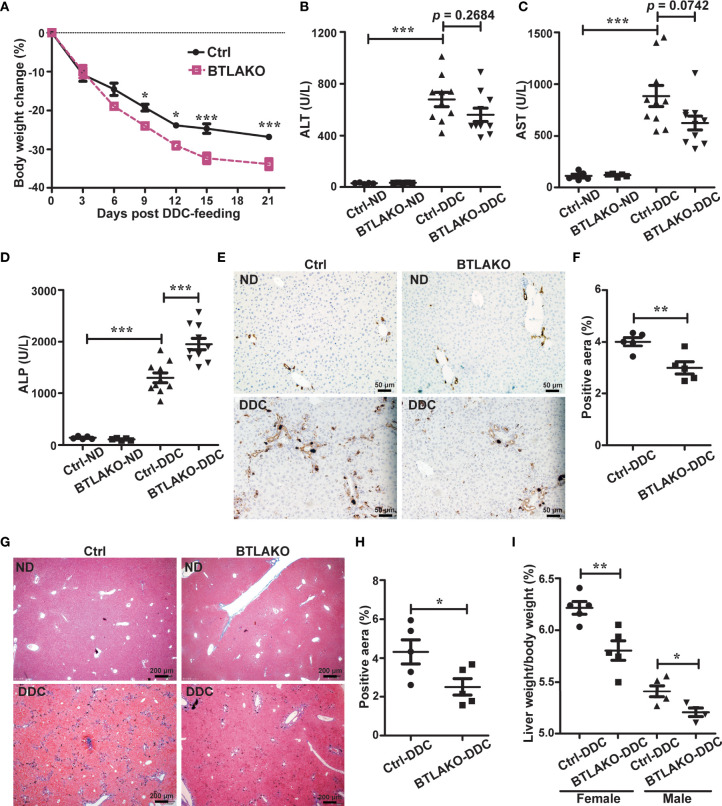
BTLA-deficiency compromises liver repair response against DDC-induced cholestatic injury. **(A)** Daily body weight changes of WT littermate controls (ctrl) and *BTLA^-/-^* mice during DDC-feeding. n = 5-10 per group. **(B-D)** Serum levels of ALT **(B)**, AST **(C)**, and ALP **(D)** in WT littermate controls (ctrl) and *BTLA^-/-^* mice that fed with either a normal chow diet (ND) or a DDC-containing diet for 21 days. n = 5-10 per group. **(E, F)** Immunohistochemistry staining of CK-19 in liver sections from the indicated mice **(E)**. The percentage of CK-19-stained positive area (brown color) per visual field was calculated accordingly **(F)**. n = 5, scale bar = 50 µm. **(G, H)** Masson’s trichrome staining for collagen fiber **(G)**. The percentage of the fibrotic area (blue color) per visual field was calculated **(H)**. n = 5, scale bar = 200 µm. **(I)** The liver-to-body weight ratios 21 days after DDC-feeding. n = 5 per group. Data were represented as the mean ± SEM. *p < 0.05; **p < 0.01; ***p < 0.001. ND, normal chow diet.

All animal work was conducted with the formal approval of the animal care committee of Xuzhou Medical University and was performed at Xuzhou Medical University’s Laboratory Animal Centre followed all the mandatory laboratory health and safety procedures. All animals received humane care and were housed in a temperature-controlled room (22 ± 2°C) and subjected to a 12-h light/dark cycle, with free access to water and food. All mice used in this study were on a C57BL/6 background. The wild-type (WT) controls used in **Figures 1**, **2**, **4**, and **6** were sibling littermates derived from heterozygotes breeding. Otherwise, we used C57BL/6 WT mice purchased from Vital River Laboratory Animal Technology (Beijing, China). The *HVEM^-/-^* and *LIGHT^-/-^* mice were kindly provided by Dr. Yang-Xin Fu (University of Texas Southwestern). The *BTLA^-/-^* mice were purchased from Biomodel Organism Science & Technology Development (Shanghai, China). Unless otherwise indicated, 6-8-week-old male mice were used in the experiments.

To induce a chronic cholestatic liver injury, mice were fed a chow diet containing 0.1% DDC (137030, Sigma-Aldrich, Darmstadt, Germany) for 3-4 weeks.

For neutrophil depletion, mice were administered intraperitoneally with 200 μg/mouse of anti-Ly6G mAb clone 1A8 (BE0075, Bio X Cell, New Hampshire, USA) on day 1 of DDC-feeding, and were then injected 100 μg/mouse twice a week until the experiment ended. Rat IgG2a (BP0089, Bio X Cell, New Hampshire, USA) was used as the isotype control.

For deoxycholic acid (DCA) or taurodeoxycholic acid (TDCA) treatments, the indicated doses of DCA (A100613, Sangon Biotech, Shanghai, China) or 40 mg/kg of TDCA (A603877, Sangon Biotech) were administered once every other day *via* oral gavage.

To deplete gut microbiota, mice received an antibiotic cocktail [1 g/L neomycin (N6386, Sigma-Aldrich), 0.5 g/L vancomycin (A600983, Sangon Biotech), 1 g/L ampicillin (A100741, Sangon Biotech), and 1 g/L metronidazole (A600633, Sangon Biotech)] in drinking water for 3 weeks. The antibiotic-containing water was changed twice a week.

### Bacterial Culture

For *Citrobacter rodentium* infection, *C. rodentium* DBS100 (ATCC51459) was grown in Luria-Bertani broth at 37°C for 15–16 h. After centrifugation and washing with PBS three times, the bacteria were suspended in PBS at a proper concentration. Mice were infected once with 1 × 10^9^ CFU/mouse by oral gavage.

For commensal bacteria culture, the cecal contents of *HVEM^+/-^* or *BTLA^+/-^* mice were resuspended in 100 times volume of sterile PBS followed by a static settlement for 10 min at room temperature. Then, 100 μl of the supernatant was inoculated anaerobically on a MRS agar plate overnight at 37°C. All the colonies formed were transferred to MRS medium and cultured for another 6-8 h in an anaerobic workstation (Don Whitley Scientific, Bingley, UK) before used for ELISA test.

### Serum Biochemistry

The serum collected from the experiments was diluted with normal saline before testing for alanine aminotransferase (ALT), aspartate aminotransferase (AST), and alkaline phosphatase (ALP) by commercially available diagnostic kits (A009, A010, A059, Nanjing Jiancheng Biological Reagent Co., Ltd., Nanjing, China).

### Histological Examinations

Sections from formalin-fixed and paraffin-embedded liver tissues were stained with a Masson’s trichrome staining kit (D026, Nanjing Jiancheng Biological Reagent Co., Ltd., Nanjing, China) to determine collagen fiber deposition.

For immunohistochemical staining, the 4% formaldehyde-fixed liver tissues were embedded in paraffin followed by sectioning, de-paraffin, rehydration, and antigen retrieval with 0.01 M sodium citrate. After treated with 3% H_2_O_2_, the samples were blocked with 5% goat serum or BSA. Then the tissue sections were incubated with anti-cytokeratin 19 (CK-19) and anti-myeloperoxidase (MPO) antibodies (ab133496 and ab9535, Abcam, Cambridge, United Kingdom) overnight. The HRP-conjugated secondary antibody (ZB-2301, ZSGB-Bio, Beijing, China) and DAB reagents were then used for visualization. As negative controls, we also stained the slides with the secondary antibody only. For quantification, we used Image J software (National Institutes of Health, Bethesda, MD, USA) to calculate antibody-stained positive area per visual field.

### Myeloperoxidase Activity Test

Myeloperoxidase activity was determined with a tissue MPO colorimetry assay kit (GMS50018, Genmed Scientifics, Shanghai, China) according to the manufacturer’s instructions.

### Flow Cytometry Analysis

Liver tissues were collected freshly and added with ice-cold PBS before being forced gently through 70-µm cell strainers. The cell suspensions were centrifuged at 40 ×*g* for 5 min at 4°C and the supernatants were transferred to new tubes followed by another centrifugation at 430 ×*g* for 5 min at 4°C. The pellets were re-suspended in 40% Percoll in PBS and centrifuged at 1,265 ×*g* with the no-brake setting for 30 min at room temperature. The supernatants were discarded and erythrocyte lysis buffer was added to the pellets containing the intrahepatic immune cells. After being washed three times with PBS containing 1% BSA, the cells were blocked with unlabeled CD16/32 antibody (14-0161-82, eBioscience, California, USA) for 15 min. The blocked cells were washed and stained for 20 min with the following fluorophore-conjugated antibodies before flow cytometry analysis: CD45-APC (561018, BD Pharmingen, New Jersey, USA), CD11b-FITC (557396, BD Pharmingen), Ly6G-PE (12-9668-82, eBioscience), Ly6C-PE Cy7 (560593, BD Pharmingen), F4/80-BV421 (565411, BD Pharmingen).

### Cytometric Bead Array

For liver Th1 and Th17 cytokines quantification, the BD™ CBA Mouse Inflammation Kit (552364, BD BioScience, New Jersey, USA) was used according to the manufacturer’s instructions.

### Bile Acids-Targeted Metabolomic Analysis

This was performed by Shanghai Biotree biomedical technology co., LTD. (Shanghai, China). Briefly, the liver and cecal content samples were suspended in 20 times volume of extraction solvent (acetonitrile-methanol-water, 2:2:1, containing 1% formic acid and 50 nmol/L internal standards), then were vortexed for 30 s, homogenized at 45 Hz for 4 min, and sonicated for 5 min in an ice-water bath. After three-time repetition of homogenization and sonication, the samples were incubated at -20°C for 1 h followed by a 15 min centrifugation at 12000 ×g at 4°C. The resulting supernatants were diluted 5 times and transferred to LC-MS vials and stored at -80°C until the UHPLC-QE Orbitrap/MS analysis. The serum samples were mixed with 4 times volume of extraction solvent (acetonitrile-methanol, 1:1, containing 1% formic acid), then were vortexed for 30 s, sonicated for 5 min in an ice-water bath followed by an incubation of -20°C for 60 min and centrifugation at 12000×g, 4°C for 15 min. The clear supernatants were transferred to an auto-sampler vial for UHPLC-MS/MS analysis.

The UHPLC separation was carried out using an Agilent 1290 Infinity II series UHPLC System (Agilent Technologies, Santa Clara, CA, USA), equipped with a Waters ACQUITY UPLC BEH Amide column (150 × 2.1 mm, 1.7 μm, Waters, Milford, MA, USA). The mobile phase A was 10 mmol/L ammonium formate and 0.1% formic acid in water, and the mobile phase B was 0.01% formic acid in acetonitrile. The column temperature was set at 35°C. The auto-sampler temperature was set at 4°C, and the injection volume was 1 μL. An Agilent 6460 triple quadrupole mass spectrometer (Agilent Technologies, Santa Clara, CA, USA), equipped with an AJS electrospray ionization interface, was applied for assay development. Typical ion source parameters were: capillary voltage = +4000/-3500 V, Nozzle Voltage = +500/-500 V, gas (N2) temperature = 300°C, gas (N2) flow = 5 L/min, sheath gas (N2) temperature = 250°C, sheath gas flow = 11 L/min, nebulizer = 45 psi.

### ELISA

Cecal contents were suspended in 10 times volumes of PBS supplemented with protease inhibitor cocktail (P1010, Beyotime Biotechnology, Nantong, China) and centrifuged 12000 ×g at 4°C, supernatants were then harvested for IgA tests. Total IgA was quantified with Mouse IgA ELISA Kit (hj-C9540, Lanpai Bio, Shanghai, China) according to the manufacturer’s instructions. For detection of IgAs against bacteria, *C. rodentium* or commensal bacteria were suspended in PBS containing 1% BSA with a final concentration of 10^9^ CFU/ml, added to 96-well polystyrene plates, and air-dried at 37°C overnight. The next day, 100 µl of 80% acetone was added to each well and dried at 37°C. After washed with PBST three times, plates were then blocked with 3% BSA in PBS for 1 h at room temperature. 50 μl supernatants from the cecal contents were then three times serially diluted and added to the wells, and incubated for 1 h at 37°C. The plate was then washed three times with PBST, and the 1: 10000 diluted HRP-linked anti-mouse IgA antibody (ab97235, Abcam, Cambridge, UK) was added, incubated for another 1 h at 37°C. Finally, an HRP substrate was added for colorimetric determination of the relative quantity of cognate IgA.

### Western Blot Analysis

Tissues or cells were homogenized in RIPA lysis buffer that contained 50 mM Tris (pH 7.4), 150 mM NaCl, 1% Triton X-100, 1% sodium deoxycholate, 0.1% SDS with freshly added protease inhibitors (P1010, Beyotime Biotechnology, Nantong, China) and phosphatase inhibitor (P1097, Beyotime Biotechnology, Nantong, China). The extract was centrifuged at 12000 ×g for 10 min at 4°C. Supernatants were collected and analyzed with bicinchoninic acid (P0010, Beyotime Biotechnology, Nantong, China) to quantify protein concentration. Proteins were resolved by SDS-PAGE and transferred to PVDF membranes. The rabbit polyclonal Ab against HVEM (Ab47677, Abcam, Cambridge, UK) and β-Actin (Ac038, Abclonal, Wuhan, China). Band intensities were quantified with ImageJ software.

### Quantitative PCR Analysis

DNA was extracted from fecal pellets (60-120 mg per sample) using the TIANamp Stool DNA Kit (DP328, Tiangen Biotech, Beijing, China) according to the manufacturer’s instructions. Total RNA was extracted from tissues homogenized in Trizol (15596018, Thermo Fisher Scientific, Waltham, USA). 1 μg of purified RNA was used to generate cDNA with a High-Capacity cDNA Reverse Transcription Kit (RR047A, Takara, Dalian, China). The qPCR reaction was performed using the AceQ qPCR SYBR Green Master Mix kit (Q111, Vazyme, Nanjing, China). The relative mRNA expression levels and the BSH positive bacteria abundance were all determined by the 2^-ΔΔCt^ method with *β-Actin* or total bacterial *16S* rDNA as the internal reference controls, respectively. Primer sequences are listed in **Supplementary Table S1**.

### Statistical Analysis

Data were analyzed with Prism (GraphPad Software, San Diego, CA, USA) and are presented as means ± SEM. Statistical significance was determined using the unpaired 2-tailed Student’s t-test between two groups for a single variable with normal distributions, one-way ANOVA with Tukey’s *post hoc* tests for three or more groups at a single time point, or two-way ANOVA followed by Bonferroni *post hoc* tests for two or more groups with two independent variables. Values of *p*<0.05 were statistically significant.

## Results

### HVEM-Deficiency Compromises Hepatic Repair to a Chemical-Induced Chronic Cholestatic Liver Injury

To study a possible role of HVEM in cholestatic liver disease, we established a chemical-induced chronic cholestatic liver injury model by feeding mice with DDC in diet (10, 11). HVEM expression in the liver was significantly increased after DDC-feeding in WT B6 mice (**Supplementary Figure S1**), suggesting that HVEM might involve in the pathogenesis of cholestasis. To further characterize the biological function of HVEM in this process, we fed *HVEM^-/-^* mice with a DDC-supplemented diet. *HVEM^-/^* ^-^ mice showed more body weight loss during the period of DDC feeding (**Figure 1A**). The serum levels of alanine transaminase (ALT) and alkaline phosphatase (ALP) were also increased more in *HVEM^-/-^* mice than WT littermate control mice, although the serum aspartate aminotransferase (AST) levels were increased indistinctively between the two groups (**Figures 1B–D**).

A cholestatic liver injury will unavoidably activate hepatic repair responses including bile duct remodeling (also called ductular reaction), fibrosis, and other forms of responses (12, 13). We, therefore, examined the ductular reaction by immunohistochemical staining for cytokeratin 19 (CK-19) and the fibrotic response by Masson’s staining for collagen fiber. Compared to the control mice, *HVEM^-/-^* mice exhibited milder ductular reaction and periductular fibrosis, suggesting weaker repair responses (**Figures 1E–H**). In further support of this notion, the liver-to-body weight ratios of *HVEM^-/-^* mice were also smaller after 21 days of DDC-feeding (**Figure 1I**). Collectively, the data suggest that HVEM plays a protective role in cholestatic liver injury.

### BTLA Is Required to Protect From DDC-Induced Cholestatic Liver Injury

HVEM has multiple ligands, including LIGHT and BTLA (8). The transcriptional levels of both *Light* and *Btla* in the liver were upregulated upon DDC-feeding in the WT mice (**Supplementary Figure S1A**), suggesting that both LIGHT and BTLA might participate in DDC-induced hepatic responses. To determine which one is responsible for interacting with HVEM to protect against the DDC-induced cholestatic liver injury, *LIGHT^-/-^* and *BTLA^-/-^* mice were challenged with DDC in the diet. In contrast to *HVEM^-/-^* mice, *LIGHT^-/-^* mice exhibited similar levels of body weight loss and liver damage after the DDC challenge when compared with WT littermate controls (**Supplementary Figure S2**). These ruled out LIGHT as the ligand that interacts with HVEM to protect mice from cholestatic liver injury.

In contrast to *LIGHT^-/-^*, *BTLA^-/-^* mice showed similar phenotypes as *HVEM^-/-^* mice upon DDC challenge. Like *HVEM^-/-^* mice, *BTLA^-/-^* mice had more body weight loss during the period of DDC feeding compared with WT littermate controls (**Figure 2A**). The serum ALP levels were increased more in the *BTLA^-/-^* mice compared with controls, although the ALT/AST levels were increased similarly (**Figures 2B–D**). Furthermore, *BTLA^-/-^* mice also exhibited milder ductular reaction, milder periductular fibrosis, and smaller liver-to-body weight ratios, suggesting weaker repair responses (**Figures 2E–I**). These findings suggest the HVEM-BTLA axis may restrain at least partially the DDC-induced cholestatic hepatobiliary injury.

### The HVEM-BTLA Axis Promotes the Increase in Hepatic Neutrophils During a Cholestatic Liver Injury

The pathophysiological process of chronic cholestatic liver disease often involves immune cell activation mediated by T-helper 1 (Th1) and T-helper 17 (Th17) cells *via* signaling through IL-12/IL-23, which induces secretion of interferon-γ and IL-17 (14). However, hepatic cytokine levels of IFN-γ, TNF, IL12p70, IL6, and IL10 did not show a significant difference between WT and *HVEM^-/-^* mice after DDC treatment (**Supplementary Figure S3A**). In addition, the transcripts of *Il17a* and *IL23* were expressed similarly between WT and *HVEM^-/-^* mice (**Supplementary Figure S3B**).

**Figure 3:**
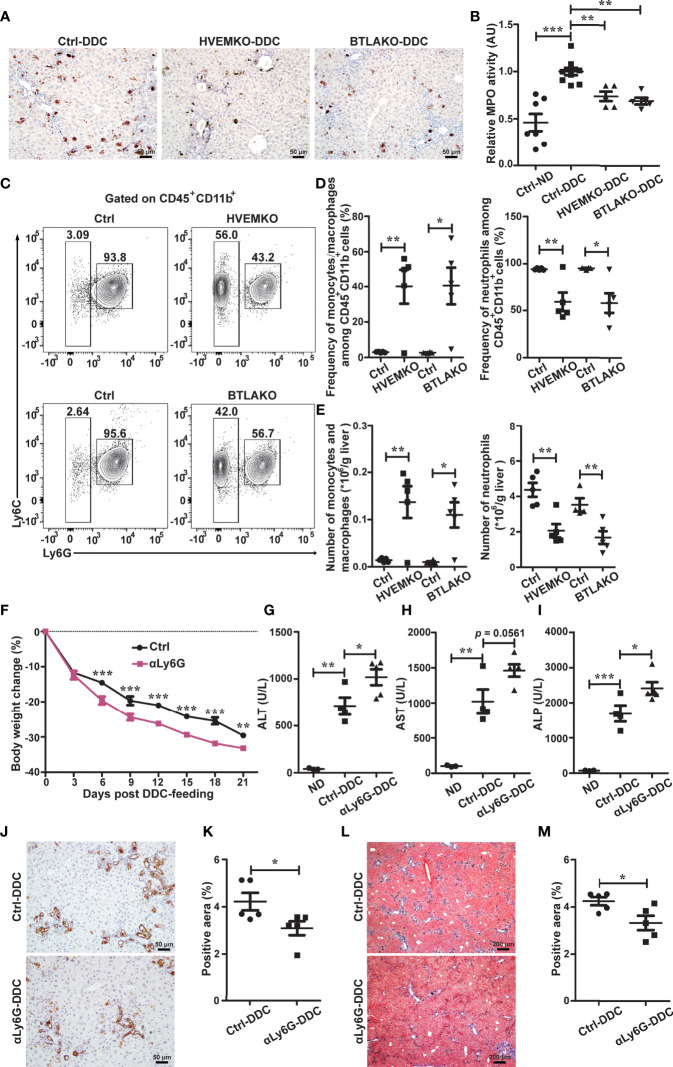
The HVEM-BTLA axis promotes the increase in hepatic neutrophils, which is essential for protection from cholestatic liver injury. **(A)** Immunohistochemistry staining of MPO in liver sections from WT controls (ctrl), *HVEM^-/-^*, and *BTLA^-/-^* mice after 21 days of DDC-feeding. n = 5, scale bar = 50 µm. **(B)** MPO activity tests for liver tissues from the indicated mice that were fed with either a normal chow diet (ND) or a DDC-containing diet for 21 days. n = 5-8 per group. **(C–E)** Representative FACS contour plot **(C)** and graph of percentage **(D)** and absolute number **(E)** of neutrophils (CD45^+^CD11b^+^Ly6G^+^) and monocyte/macrophage subset (CD45^+^CD11b^+^Ly6G^-^Ly6C^hi-lo^) in livers of WT controls (ctrl), *HVEM^-/-^*, and *BTLA^-/-^* mice after 21 days of DDC-feeding. n = 4-5 per group. **(F)** Daily body weight changes of WT mice that were treated with a control antibody (ctrl) or anti-Ly6G during DDC-feeding. n = 5 per group. **(G–I)** Serum ALT **(G)**, AST **(H)**, and ALP **(I)** levels of the indicated mice after 21 days of DDC-feeding. n = 3-5 per group. (J and K) Immunohistochemistry staining of CK-19 in liver sections from the indicated mice **(J)**. The percentage of CK-19-stained positive area (brown color) per visual field was calculated accordingly **(K)**. n = 5, scale bar = 50 µm. **(L, M)** Masson’s trichrome staining for collagen fiber (L). The percentage of the fibrotic area (blue color) per visual field was calculated (M). n = 5, scale bar = 200 µm. Data were represented as the mean ± SEM. *p < 0.05; **p < 0.01; ***p < 0.001.

Recent work has implied that myeloid cells, especially neutrophils, have anti-inflammatory functions and contribute to liver repair after acute injury (15). We wondered whether the HVEM-BTLA axis regulates intrahepatic myeloid cells during chronic cholestatic liver injury. Therefore, we performed immunohistochemical staining and activity test for MPO, a heme protein that constitutes the major component of neutrophil azurophilic granules. Both *HVEM^-/-^* and *BTLA^-/-^* mice had a significant reduction of the post-injury-induced increment of hepatic MPO-positive myeloid cells (**Figures 3A, B**), suggesting the HVEM-BTLA axis might promote intrahepatic MPO-positive myeloid cell accumulation during liver damage.

To better define the myeloid cell population, we performed multiparameter flow cytometry analysis on purified non-parenchymal liver cells. Under homeostatic conditions, there were normal numbers of neutrophils (defined as CD45^+^CD11b^+^Ly6G^+^), monocyte/macrophage subsets (CD45^+^CD11b^+^Ly6G^-^Ly6C^hi-lo^), and mature recruited macrophages (CD45^+^CD11b^+^Ly6G^-^Ly6C^hi-lo^F4/80^+^) in *HVEM^-/-^* and *BTLA^-/-^* mice (**Supplementary Figure S4**). 21 days after DDC treatment, both the percentage and absolute numbers of neutrophils were reduced in *HVEM^-/-^* and *BTLA^-/-^* mice (**Figures 3C–E**), consistent with the MPO staining pattern. In compensation for that, the intrahepatic monocyte/macrophage subsets were increased (**Figures 3C–E**). However, the absolute numbers of mature recruited macrophages remained the same (**Supplementary Figure S5**).

### Antibody-Mediated Neutrophil Depletion Worsens DDC-Induced Cholestatic Hepatobiliary Injury

To investigate the role of neutrophils in DDC-induced cholestatic liver injury, we administered a neutrophil-depleting antibody, anti-Ly6G, to WT B6 mice. As expected, anti-Ly6G treatment effectively reduced the number of neutrophils in the liver (**Supplementary Figure S6**). Concomitantly it resulted in exacerbation of liver damage and reduction of ductular reaction and periductular collagen deposition (**Figures 3F–M**).

### HVEM-Deficient Mice Have a Reduced Ability to Deconjugate Bile Acids During DDC-Feeding

The infiltration of neutrophils into tissue requires chemokine/chemokine receptor interaction. We, therefore, determined the hepatic expression of genes related to neutrophil chemotaxis in *HVEM^-/-^* and *BTLA^-/-^* mice after DDC-feeding. Their levels were almost indistinguishable from those in the WT control mice (**Supplementary Figure S7**).

**Figure 4:**
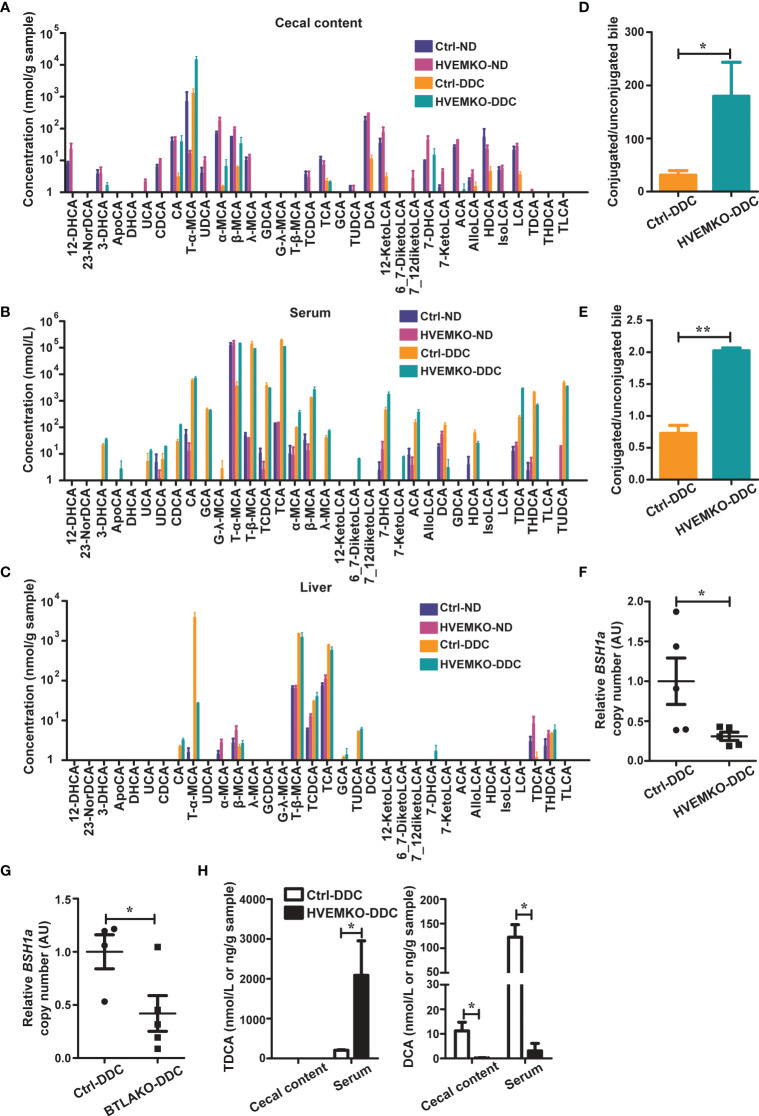
HVEM-deficient mice have a reduced ability to deconjugate bile acids during DDC-feeding. **(A–C)** The bile acids profiles in the cecal contents **(A)**, serum **(B)**, and liver tissues **(C)** from WT controls (ctrl) and *HVEM^-/-^* mice that fed with a normal chow diet (ND) or a DDC-containing diet for 21 days. n = 3-5. **(D, E)** The ratio between conjugated and unconjugated bile acids in the cecal contents **(D)** and the serum **(E)** of the indicated mice after 21 days of DDC-feeding. **(F, G)** Relative DNA copy number of bsh1 gene belonging to the BSH group 1A bacteria in the cecal contents of *HVEM^-/-^* **(F)** and *BTLA^-/-^* **(G)** mice after 21 days of DDC-feeding, estimated by qPCR. n = 4-5. **(H)** The TDCA and DCA concentrations in the serum and cecal contents of controls and *HVEM^-/-^* mice after 21 days of DDC-feeding. n = 3-5 per group. Data were represented as the mean ± SEM. *p < 0.05; **p < 0.01.

It was reported that the secondary bile acid deoxycholic acid (DCA) could increase neutrophils by expanding bone marrow granulocyte-monocyte progenitors, and taurodeoxycholate (TDCA) could increase the number of myeloid-derived suppressor cells (MDSCs) (16, 17). These promoted us to determine the bile acid profiles of *HVEM^-/-^* mice. We first examined the expression of genes that involve in bile acids synthesis, transport, and signaling. The genes we tested were no differences between the controls and *HVEM^-/-^* mice after DDC-feeding (**Supplementary Figure S8**). We then performed bile acid profiling on serum, liver, and cecal contents of the WT controls and *HVEM^-/-^* mice before and after DDC-feeding. There were only subtle differences in the bile acid profiles between the two strains of mice before DDC-feeding, but the differences became more obvious after the DDC challenge (**Figures 4A–C**). Noticeably, the ratios between the conjugated to the unconjugated bile acids were increased in both the cecal contents and the serum of *HVEM^-/-^* mice after the DDC challenge (**Figures 4D, E**), implying that during cholestasis *HVEM^-/-^* deficiency might exacerbate dysbiosis-induced reduction of bile acid biotransformation by the microbial-derived deconjugation enzyme bile salt hydrolases (BSHs) (18). In agreement with this, the relative abundance of BSH-producing organisms that belongs to members of *bsh* group 1A was reduced in *HVEM^-/-^* as well as *BTLA^-/-^* mice compared to their control mice after the DDC challenge (**Figures 4F, G**, and **Supplementary Figure S9**), according to qPCR analysis of relative DNA copy number of the corresponding *bsh* genes. Furthermore, the conjugated secondary bile acid TDCA was accumulated more in the serum of *HVEM^-/-^* mice, while its deconjugated-form DCA was diminished in both the cecal content and the serum compared to the WT controls (**Figure 4H**).

### TDCA Increases Intrahepatic Neutrophils to Protect From Cholestatic Liver Injury

Considering that DCA was reported to increase neutrophils (16), we tested the effect of DCA in DDC-induced cholestatic liver injury and found that DCA had no protective effect (**Supplementary Figure S10**).

**Figure 5:**
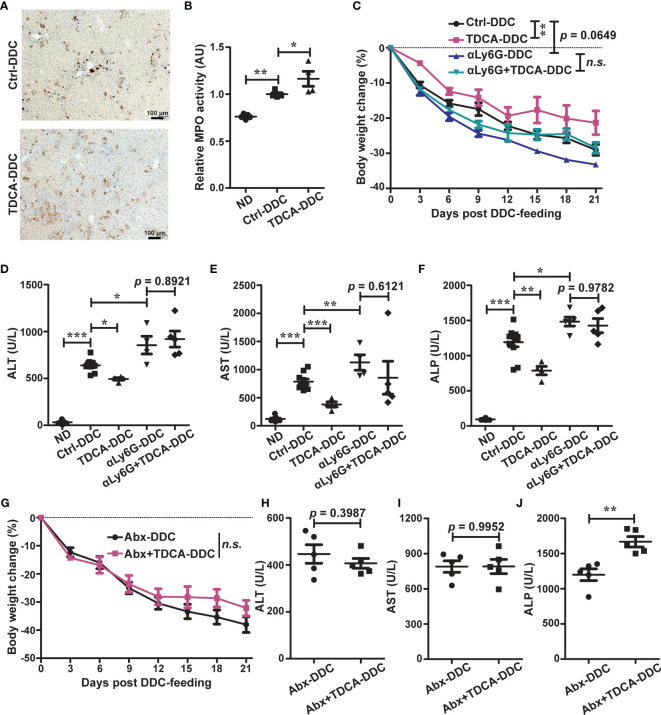
TDCA increases intrahepatic neutrophils to protect against cholestatic liver injury in a gut microbiota-dependent manner. **(A, B)** Immunohistochemistry staining **(A)** and activity tests **(B)** of liver MPO from WT mice that were treated with vehicle (ctrl) or TDCA and in the meantime fed with a normal chow diet or a DDC-containing diet for 21 days. n = 4. **(C)** Daily body weight changes of WT mice that were treated with vehicle (ctrl), TDCA, anti-Ly6G, or TDCA plus anti-Ly6G. n = 4-10. **(D–F)** Serum ALT, AST, and ALP levels in WT mice that were treated with vehicle (ctrl), TDCA, anti-Ly6G, or TDCA plus anti-Ly6G, and in the meantime fed with a normal chow diet or a DDC-containing diet for 21 days. n = 4-10. **(G–J)** WT B6 mice were treated with vehicle (ctrl) or TDCA *via* oral gavage, together with a cocktail of broad-spectrum antibiotics (Abx) in drinking water for 21 days. **(G)** Daily body weight changes. **(H–J)** Serum levels of ALT **(H)**, AST **(I)** and ALP **(J)**. n = 5 per group. Data were represented as the mean ± SEM. *p < 0.05; **p < 0.01; ***p < 0.001; ns, no statistical significance.

We then tested TDCA. TDCA treatment in WT mice during DDC-feeding increased intrahepatic MPO-positive cells (**Figures 5A, B**). TDCA treatment further improved cholestatic liver injury (**Figures 5C–F**), and this effect was correlated with neutrophils, as depletion of neutrophils by anti-Ly6G counteracted the protective effect of TDCA (**Figures 5C–F**).

### TDCA Requires Further Biotransformation by the Gut Microbiota to Protect From DDC-Induced Injury

Since *HVEM^-/-^* mice accumulated more TDCA in the serum than WT mice during DDC-feeding but were still more sensitive to cholestatic liver damage, we wondered why it was the case. We found that actually the effect of TDCA to improve liver damage and increase intrahepatic neutrophils still requires the gut microbiota, as depletion of the gut microbiota by antibiotics eliminated this effect (**Figures 5G–J**, and **Supplementary Figure S11**). Collectively, these findings suggest that the HVEM-BTLA axis might influence the gut microbiota to process bile acids, which promote neutrophil accumulation in the liver during DDC-feeding.

### The HVEM-BTLA Axis Regulates Mucosal IgA Responses

**Figure 6:**
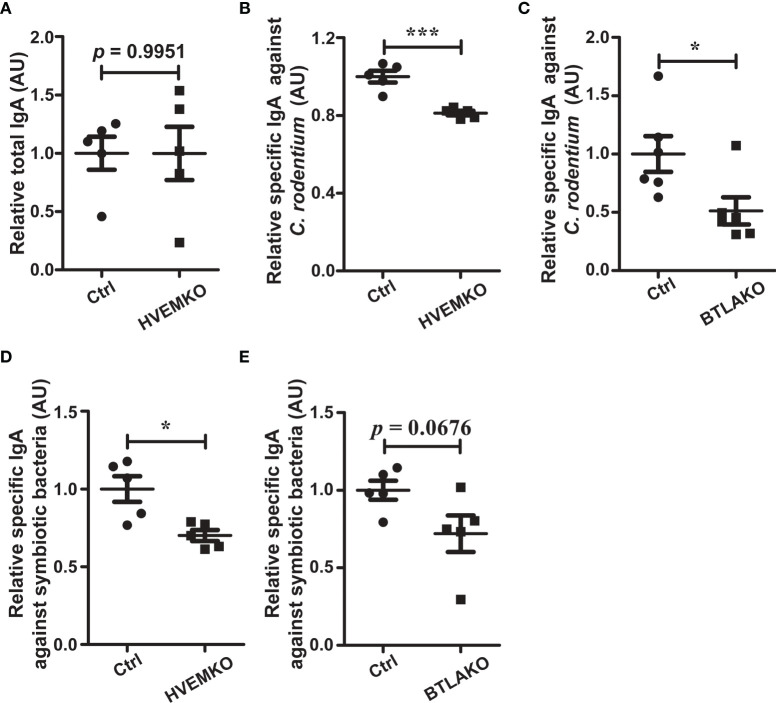
The BTLA-HVEM axis regulates mucosal IgA responses. **(A–C)** Mice were infected with *C rodentium via* oral gavage. **(A)** Total SIgA level in the cecal contents from *HVEM^-/-^* mice 10 days after *C rodentium* infection relative to that of WT littermate controls (ctrl). n = 5. **(B)** *C rodentium-*reactive SIgA level in the cecal contents from *HVEM^-/-^* mice 10 days after *C rodentium* infection relative to that of WT littermate controls (ctrl). n = 5. **(C)** *C rodentium-*reactive SIgA level in the cecal contents from *BTLA^-/-^* mice 10 days after *C rodentium* infection relative to that of WT littermate controls (ctrl). n = 5. **(D, E)** Mice were under the normal homeostatic conditions without pathogen challenge. Commensal-reactive SIgA level in the cecal contents of *HVEM^-/-^* **(D)** *or BTLA^-/-^* **(E)** mice relative to that of WT littermate controls (ctrl) were determined, respectively. n = 5. Data were represented as the mean ± SEM. * *p* < 0.05; *** *p* < 0.001.

We then questioned how the HVEM-BTLA axis might regulate the gut microbiota. It has been reported that BTLA-HVEM signaling regulates germinal center reaction (19), but whether it regulates intestinal IgA responses is not known. We, therefore, challenged *HVEM^-/-^* and *BTLA^-/-^* mice with an enteropathogen *Citrobacter rodentium.* 10-15 days after infection, we checked the intestinal IgA production. Although the level of total secretory IgA (SIgA) was not different between *HVEM^-/-^* mice and their littermate controls, the level of *C. rodentium*-reactive SIgA was decreased in *HVEM^-/-^* mice (**Figures 6A, B**). The same thing was true for *BTLA^-/-^* mice (**Figure 6C**). Furthermore, even under the normal homeostatic conditions without pathogen challenge *HVEM^-/-^* mice already had reductions of SIgA for select commensal bacteria from *in vitro* culture (**Figure 6D**), and *BTLA^-/-^* mice had a trend toward reduction (**Figure 6E**). This was correlated with a reduced gut microbiota diversity in naïve *HVEM^-/-^* mice (**Supplementary Figure S12**). On the basis of these data, we speculated that the HVEM-BTLA axis might influence the gut microbiota landscape at least in part by regulating mucosal IgA response.

## Discussion

The above findings establish that the HVEM signaling, as one of the immune checkpoints, acts to restrain a chemical-induced chronic cholestatic liver injury. *HVEM^-/-^* mice had defects in the biotransformation of bile acids by the gut microbiota during cholestasis, which affected hepatic neutrophil accumulation and compromised liver repair. The HVEM-BTLA signaling influences the gut microbiota probably at least in part by regulating mucosal IgA. We observed a slight decrease of mucosal IgA production in both *HVEM^-/-^* and *BTLA^-/-^* mice, which may be related to a role of the HVEM-BTLA signaling in the germinal center reaction by restraining low-affinity B cells from the competition with high-affinity B cells for T cell help during (19). Mucosal IgA promotes the survival of commensal bacteria while inhibiting the invasion by pathogens (20–23). In cholestasis, impaired bile flow leads to a reduction of bile acids in the gut, causing dysbiosis and a decrease of the secondary bile acid pool. A slight mucosal IgA deficiency in *HVEM^-/-^* mice may attenuate the protective role of IgA on the microbiome and further reduce the gut microbiota diversity and exacerbate the deficit of bile acids biotransformation during cholestasis, leading to weaker liver repair.

The role of bile acids in cholestatic liver diseases is dichotomous. On one hand, bile acid accumulation inside the liver induces hepatobiliary injury and promotes inflammation; On the other hand, some bile acids (e.g., ursodeoxycholic acid) are effective in treating at least partially cholestatic liver diseases (24). The same thing is true for the gut microbiota. In *Abcb4^−/−^* mice, the intestinal microbiota protects from hepatobiliary disease (25). In contrast, in NOD.c3c4 mice, the lack of microbes has a protective effect on bile duct disease progression (26). These differences highlight the complexity of the gut-liver axis, which requires further investigation.

It should be mentioned that although we claimed that bile acid protection from cholestasis in our model was associated with increased hepatic neutrophils, there may be heterogeneity within this population that requires further study. Since neutrophils and MDSCs share markers and also likely ontogeny, and the anti-Ly6G antibody will likely deplete both cell populations, which type of cells are responsible for liver protection during DDC-induced cholestatic injury remains to be determined. Neutrophils are generally thought to exacerbate tissue injury by releasing proteases and oxidants, but they are also involved in tissue repair programs (15). For example, neutrophils can generate a number of important anti-inflammatory and pro-resolving lipid mediators (27), and they can also coordinate with macrophages to trigger the resolution of tissue damage (15).

Furthermore, the two antibodies currently available to deplete neutrophils in mice, namely anti-Ly6G clone 1A8 and anti-Gr1 clone RB6-8C5, suffer some limitations, and data obtained by using these two antibodies should be interpreted carefully. Obviously from our data, the anti-Ly6G treatment has an effect, but whether this is due to real depletion of neutrophils or rather altered functionality of neutrophils remains to be determined. New tools are definitely needed in order to better understand the *in vivo* role of neutrophils.

A previous study indicated that administration of DCA intravenously expanded bone marrow granulocyte-monocyte progenitors and intestinal neutrophils and protected from amoebic colitis (16). In our study, we found that TDCA was instead more effective in expanding hepatic neutrophils/MDSCs and was more hepatoprotective during cholestasis. This is consistent with the role of TDCA in expanding anti-inflammatory MDSCs during sepsis (17). Compared to DCA, whether TDCA is more likely to trigger a tissue repair program remains to be investigated. Furthermore, the effect of TDCA on neutrophils/MDSCs was dependent on downstream microbial transformation, as antibiotics treatment diminished this effect. Since TDCA can be transformed not only to DCA but also to other secondary bile acids, such as isoDCA (28), it remained to be determined which secondary bile acid actually promotes the neutrophil/MDSC expansion. In any case, the relationship between bile acids and neutrophils/MDSCs is influenced by the gut microbiota. In addition, bile acids can also regulate adaptive immune responses by directly modulating the balance of T helper 17 cells and regulatory T cells (29, 30), suggesting much broader roles of bile acids in modulating inflammation and tissue repair.

Despite current immunosuppression therapy by glucocorticoids being ineffective to treat cholestasis, cholestatic liver diseases are still thought to be chronic immune-mediated biliary diseases (24, 31). The cell surface co-stimulatory molecule CD28, which is necessary for T-cell activation, survival, and proliferation, is recognized as a risk factor in PSC (32), suggesting immune checkpoint networks may regulate cholestatic inflammation. Our data further support this notion and even add the HVEM-BTLA axis into the picture. Immune response during cholestasis is a dynamic process, HVEM can execute both positive and negative functions during inflammation, how to specifically engage negative or positive functions of HVEM during cholestasis is worth for future exploration.

In conclusion, our data reveal the HVEM-BTLA axis as an important regulator to restrain chronic cholestatic liver injury. Modulation of immune checkpoints represents a potential therapeutic strategy for cholestatic liver diseases as well as other inflammatory diseases.

## Data Availability Statement

The raw data supporting the conclusions of this article will be made available by the authors, without undue reservation.

## Ethics Statement

The animal study was reviewed and approved by the animal care committee of Xuzhou Medical University.

## Author Contributions

YK and XZ: collection and assembly of data, acquisition, analysis and interpretation of data, manuscript writing. LM: collection and assembly of part of data, analysis and interpretation of part of data. ML, SX, QJ, SZ, HW, JH, ZL, and YXW: collection and assembly of part of data. YGW: conception and design of data, manuscript writing, revising it critically for important intellectual content. All authors read and approved the final manuscript.

## Funding

This work was supported, in part, by the National Natural Science Foundation of China (nos: 81770853 and 81700774).

## Conflict of Interest

The authors declare that the research was conducted in the absence of any commercial or financial relationships that could be construed as a potential conflict of interest.

## Publisher’s Note

All claims expressed in this article are solely those of the authors and do not necessarily represent those of their affiliated organizations, or those of the publisher, the editors and the reviewers. Any product that may be evaluated in this article, or claim that may be made by its manufacturer, is not guaranteed or endorsed by the publisher.

## References

[1] Trauner M Boyer JL. Cholestatic Syndromes. Curr Opin Gastroenterol (2004) 20(3):220–30. doi: 10.1097/00001574-200405000-00006 15703646

[2] Hirschfield GM Heathcote EJ Gershwin ME. Pathogenesis of Cholestatic Liver Disease and Therapeutic Approaches. Gastroenterology (2010) 139(5):1481–96. doi: 10.1053/j.gastro.2010.09.004 20849855

[3] Copple BL Jaeschke H Klaassen CD. Oxidative Stress and the Pathogenesis of Cholestasis. Semin Liver Dis (2010) 30(2):195–204. doi: 10.1055/s-0030-1253228 20422501

[4] Guicciardi ME Trussoni CE Krishnan A Bronk SF Lorenzo Pisarello MJ O’Hara SP. Macrophages Contribute to the Pathogenesis of Sclerosing Cholangitis in Mice. J Hepatol (2018) 69(3):676–86. doi: 10.1016/j.jhep.2018.05.018 PMC6098983 29802947

[5] Tedesco D Thapa M Chin CY Ge Y Gong M Li J. Alterations in Intestinal Microbiota Lead to Production of Interleukin 17 by Intrahepatic Gammadelta T-Cell Receptor-Positive Cells and Pathogenesis of Cholestatic Liver Disease. Gastroenterology (2018) 154(8):2178–93. doi: 10.1053/j.gastro.2018.02.019 PMC5985208 29454797

[6] Wang L Sun Y Zhang Z Jia Y Zou Z Ding J. CXCR5+ CD4+ T Follicular Helper Cells Participate in the Pathogenesis of Primary Biliary Cirrhosis. Hepatology (2015) 61(2):627–38. doi: 10.1002/hep.27306 PMC4507804 25042122

[7] Steinberg MW Cheung TC Ware CF. The Signaling Networks of the Herpesvirus Entry Mediator (TNFRSF14) in Immune Regulation. Immunol Rev (2011) 244(1):169–87. doi: 10.1111/j.1600-065X.2011.01064.x PMC3381650 22017438

[8] Ward-Kavanagh LK Lin WW Sedy JR Ware CF. The TNF Receptor Superfamily in Co-Stimulating and Co-Inhibitory Responses. Immunity (2016) 44(5):1005–19. doi: 10.1016/j.immuni.2016.04.019 PMC4882112 27192566

[9] Murphy KM Nelson CA Sedy JR. Balancing Co-Stimulation and Inhibition With BTLA and HVEM. Nat Rev Immunol (2006) 6(9):671–81. doi: 10.1038/nri1917 16932752

[10] Fickert P Stoger U Fuchsbichler A Moustafa T Marschall HU Weiglein AH. A New Xenobiotic-Induced Mouse Model of Sclerosing Cholangitis and Biliary Fibrosis. Am J Pathol (2007) 171(2):525–36. doi: 10.2353/ajpath.2007.061133 PMC1934539 17600122

[11] Yokoo H Harwood TR Racker D Arak S. Experimental Production of Mallory Bodies in Mice by Diet Containing 3,5-Diethoxycarbonyl-1,4-Dihydrocollidine. Gastroenterology (1982) 83(1 Pt 1):109–13. doi: 10.1016/S0016-5085(82)80293-X 6176493

[12] Rodrigo-Torres D Affo S Coll M Morales-Ibanez O Millan C Blaya D. The Biliary Epithelium Gives Rise to Liver Progenitor Cells. Hepatology (2014) 60(4):1367–77. doi: 10.1002/hep.27078 PMC4410184 24700364

[13] Mariotti V Strazzabosco M Fabris L Calvisi DF. Animal Models of Biliary Injury and Altered Bile Acid Metabolism. Biochim Biophys Acta Mol Basis Dis (2018) 1864(4 Pt B):1254–61. doi: 10.1016/j.bbadis.2017.06.027 PMC5764833 28709963

[14] Shah RA Kowdley KV. Current and Potential Treatments for Primary Biliary Cholangitis. Lancet Gastroenterol Hepatol (2020) 5(3):306–15. doi: 10.1016/S2468-1253(19)30343-7 31806572

[15] Yang W Tao Y Wu Y Zhao X Ye W Zhao D. Neutrophils Promote the Development of Reparative Macrophages Mediated by ROS to Orchestrate Liver Repair. Nat Commun (2019) 10(1):1076. doi: 10.1038/s41467-019-09046-8 30842418 PMC6403250

[16] Burgess SL Leslie JL Uddin J Oakland DN Gilchrist C Moreau GB. Gut Microbiome Communication With Bone Marrow Regulates Susceptibility to Amebiasis. J Clin Invest (2020) 130(8):4019–24. doi: 10.1172/JCI133605 PMC7410058 32369444

[17] Chang S Kim YH Kim YJ Kim YW Moon S Lee YY. Taurodeoxycholate Increases the Number of Myeloid-Derived Suppressor Cells That Ameliorate Sepsis in Mice. Front Immunol (2018) 9:1984. doi: 10.3389/fimmu.2018.01984 30279688 PMC6153344

[18] Mullish BH McDonald JAK Pechlivanis A Allegretti JR Kao D Barker GF. Microbial Bile Salt Hydrolases Mediate the Efficacy of Faecal Microbiota Transplant in the Treatment of Recurrent Clostridioides Difficile Infection. Gut (2019) 68(10):1791–800. doi: 10.1136/gutjnl-2018-317842 PMC6839797 30816855

[19] Mintz MA Felce JH Chou MY Mayya V Xu Y Shui JW. The HVEM-BTLA Axis Restrains T Cell Help to Germinal Center B Cells and Functions as a Cell-Extrinsic Suppressor in Lymphomagenesis. Immunity (2019) 51(2):310–23.e7. doi: 10.1016/j.immuni.2019.05.022 31204070 PMC6703922

[20] Weis AM Round JL. Microbiota-Antibody Interactions That Regulate Gut Homeostasis. Cell Host Microbe (2021) 29(3):334–46. doi: 10.1016/j.chom.2021.02.009 PMC7990058 33705705

[21] Macpherson AJ Yilmaz B Limenitakis JP Ganal-Vonarburg SC. IgA Function in Relation to the Intestinal Microbiota. Annu Rev Immunol (2018) 36:359–81. doi: 10.1146/annurev-immunol-042617-053238 29400985

[22] Bunker JJ Bendelac A. IgA Responses to Microbiota. Immunity (2018) 49(2):211–24. doi: 10.1016/j.immuni.2018.08.011 PMC6107312 30134201

[23] Nakajima A Vogelzang A Maruya M Miyajima M Murata M Son A. IgA Regulates the Composition and Metabolic Function of Gut Microbiota by Promoting Symbiosis Between Bacteria. J Exp Med (2018) 215(8):2019–34. doi: 10.1084/jem.20180427 PMC6080902 30042191

[24] Lazaridis KN LaRusso NF. Primary Sclerosing Cholangitis. N Engl J Med (2016) 375(12):1161–70. doi: 10.1056/NEJMra1506330 PMC5553912 27653566

[25] Tabibian JH O’Hara SP Trussoni CE Tietz PS Splinter PL Mounajjed T. Absence of the Intestinal Microbiota Exacerbates Hepatobiliary Disease in a Murine Model of Primary Sclerosing Cholangitis. Hepatology (2016) 63(1):185–96. doi: 10.1002/hep.27927 PMC4670294 26044703

[26] Schrumpf E Kummen M Valestrand L Greiner TU Holm K Arulampalam V. The Gut Microbiota Contributes to a Mouse Model of Spontaneous Bile Duct Inflammation. J Hepatol (2017) 66(2):382–9. doi: 10.1016/j.jhep.2016.09.020 PMC5250551 27720803

[27] Buckley CD Gilroy DW Serhan CN. Proresolving Lipid Mediators and Mechanisms in the Resolution of Acute Inflammation. Immunity (2014) 40(3):315–27. doi: 10.1016/j.immuni.2014.02.009 PMC4004957 24656045

[28] Winston JA Theriot CM. Diversification of Host Bile Acids by Members of the Gut Microbiota. Gut Microbes (2020) 11(2):158–71. doi: 10.1080/19490976.2019.1674124 PMC7053883 31595814

[29] Hang S Paik D Yao L Kim E Trinath J Lu J. Bile Acid Metabolites Control TH17 and Treg Cell Differentiation. Nature (2019) 576(7785):143–8. doi: 10.1038/s41586-019-1785-z PMC6949019 31776512

[30] Campbell C McKenney PT Konstantinovsky D Isaeva OI Schizas M Verter J. Bacterial Metabolism of Bile Acids Promotes Generation of Peripheral Regulatory T Cells. Nature (2020) 581(7809):475–9. doi: 10.1038/s41586-020-2193-0 PMC7540721 32461639

[31] Lleo A Marzorati S Anaya JM Gershwin ME. Primary Biliary Cholangitis: A Comprehensive Overview. Hepatol Int (2017) 11(6):485–99. doi: 10.1007/s12072-017-9830-1 29164395

[32] Liu JZ Hov JR Folseraas T Ellinghaus E Rushbrook SM Doncheva NT. Dense Genotyping of Immune-Related Disease Regions Identifies Nine New Risk Loci for Primary Sclerosing Cholangitis. Nat Genet (2013) 45(6):670–5. doi: 10.1038/ng.2616 PMC3667736 23603763

