# Supplementary material for: The HVEM-BTLA Immune Checkpoint Restrains Murine Chronic Cholestatic Liver Injury by Regulating the Gut Microbiota
Source: Front Immunol. 2022 Feb 4;13:773341. doi: 10.3389/fimmu.2022.773341 (PMC8854854; doi:10.3389/fimmu.2022.773341)
Supplement: Supplementary file 1 [file DataSheet_1.pdf]

# *Supplementary Material*

**The HVEM-BTLA Immune Checkpoint Restrains Murine Chronic Cholestatic Liver Injury by regulating the Gut Microbiota**

Yanbo Kou, Xingping Zheng, Liyuan Meng, Mengnan Liu, Shihong Xu, Qiyue Jing, Shenghan Zhang, Hanying Wang, Jinzhi Han, Zhuanzhuan Liu, Yanxia Wei, and Yugang Wang

## 1 Supplementary Figures and Tables

### 1.1 Supplementary Figures

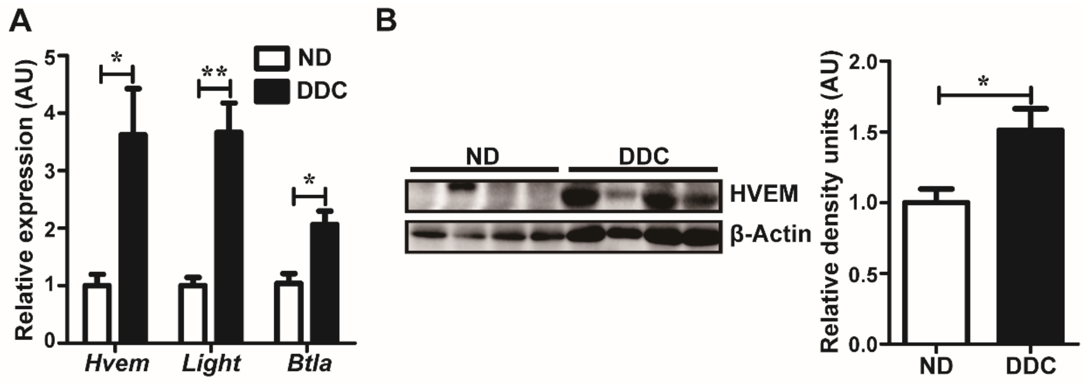


**Supplementary Figure S1. HVEM expression is increased during DDC-feeding**. **(A and B)** WT B6 mice were either fed with a normal chow diet (ND) or a DDC-containing diet for 21 days. (A) The relative mRNA levels of *Hvem*, *Light,* and *Btla* in the liver. (B) Western blot analysis of hepatic HVEM protein expression levels. n = 4. Data were represented as the mean ± SEM. * $p < 0.05$; ** $p < 0.01$.

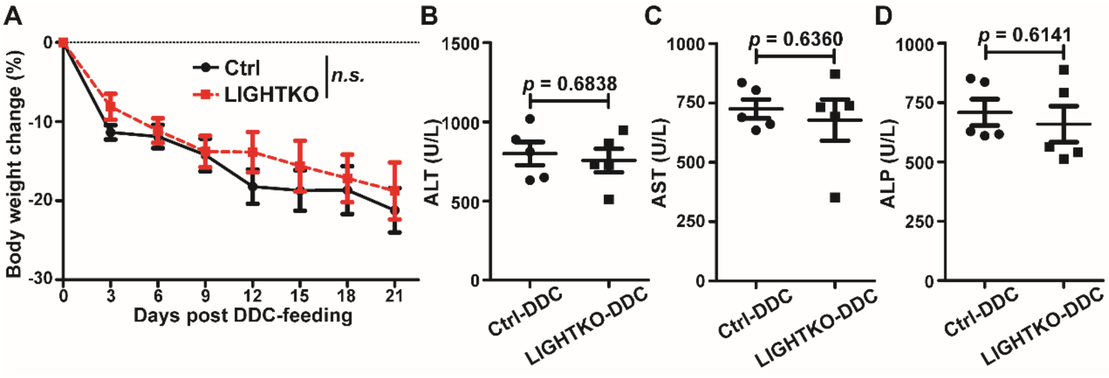


**Supplementary Figure S2. LIGHT deletion has no influence on DDC-induced cholestatic liver injury compared with the littermate controls. (A)** Bodyweight changes in *LIGHT*$^{-/-}$ and WT littermate controls (ctrl) after DDC-feeding. n = 5. **(B-D)** Serum ALT (B), AST (C), and ALP (D) levels on day 21 after DDC-feeding. ND, normal chow diet. n = 5. Data were represented as the mean ± SEM. N.S., no statistical significance.

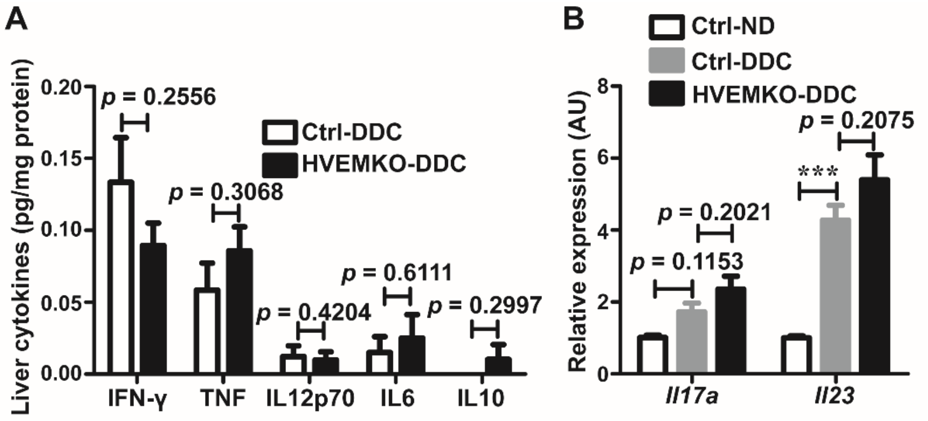


**Supplementary Figure S3. HVEM deficiency has no influence on inflammatory cytokine production during the DDC challenge compared with the controls. (A)** The hepatic protein expression levels of IFN-γ, TNF, IL12p70, IL6, and IL10 in WT controls (ctrl) and *HVEM*$^{-/-}$ mice after 3 weeks of DDC-feeding. **(B)** The relative mRNA expression levels of *Il17a* and *Il23* in the liver of the controls and *HVEM*$^{-/-}$ mice after 3 weeks of DDC-feeding. n = 3-7 per group. Data were represented as the mean ± SEM. *** $p < 0.001$.

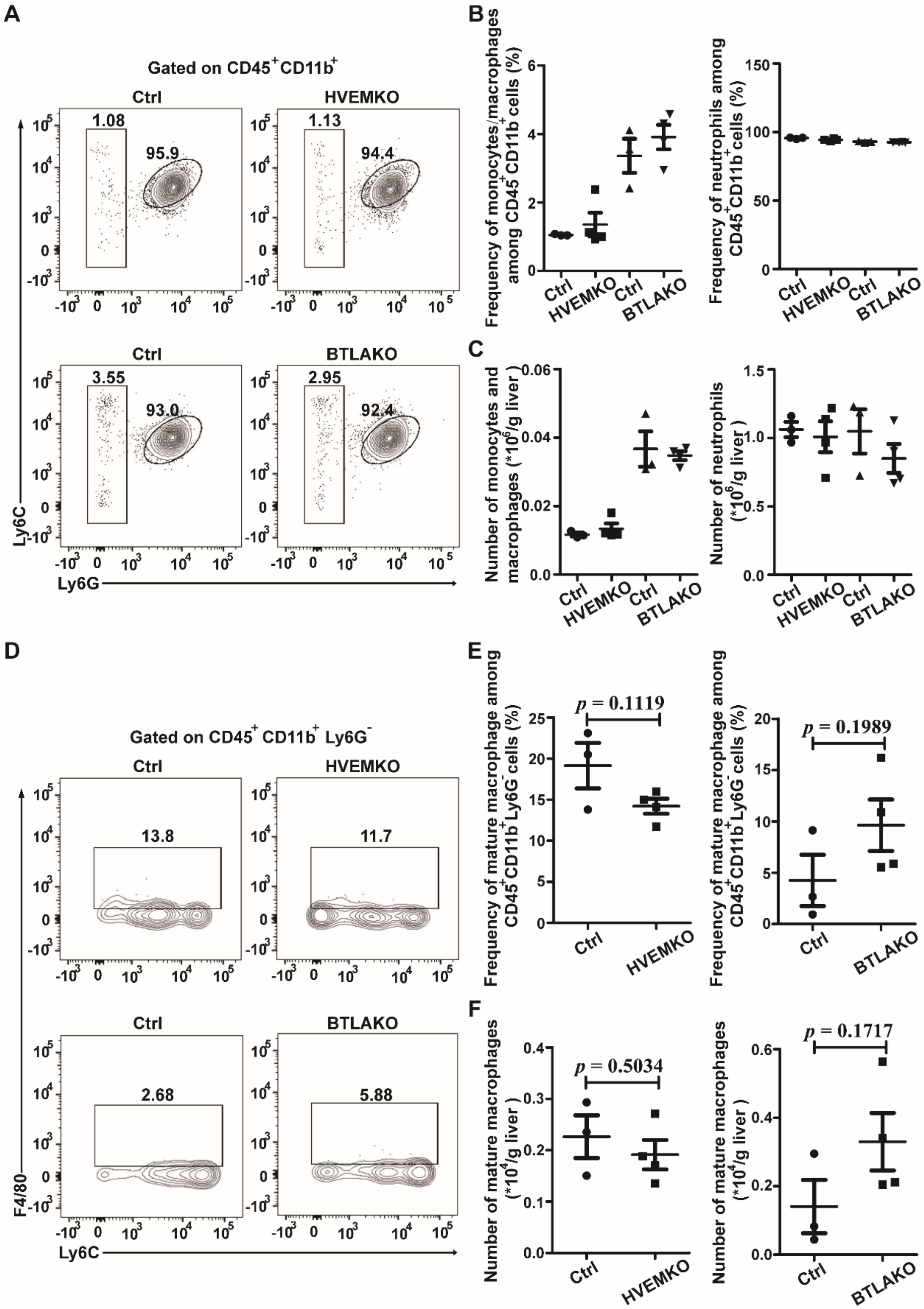


**Supplementary Figure S4. There are normal numbers of neutrophils, macrophages, and monocytes in HVEM and BTLA KOs under homeostatic conditions. (A-F)** Flow cytometry-based analysis of liver neutrophils ($CD45^+CD11b^+Ly6G^+$), monocyte/macrophage subset

($CD45^+CD11b^+Ly6G^-Ly6C^{hi\text{-}lo}$), and mature recruited macrophages ($CD45^+CD11b^+Ly6G^-F4/80^+Ly6C^{hi\text{-}lo}$) at steady state. (A and D) Representative contour plot. The percentages and numbers of liver monocyte/macrophage subsets (B and C), neutrophils (B and C), and mature recruited macrophages (E and F) from controls (ctrl), *HVEM*$^{-/-}$, and *BTLA*$^{-/-}$ mice were then determined. n = 3-4/group.

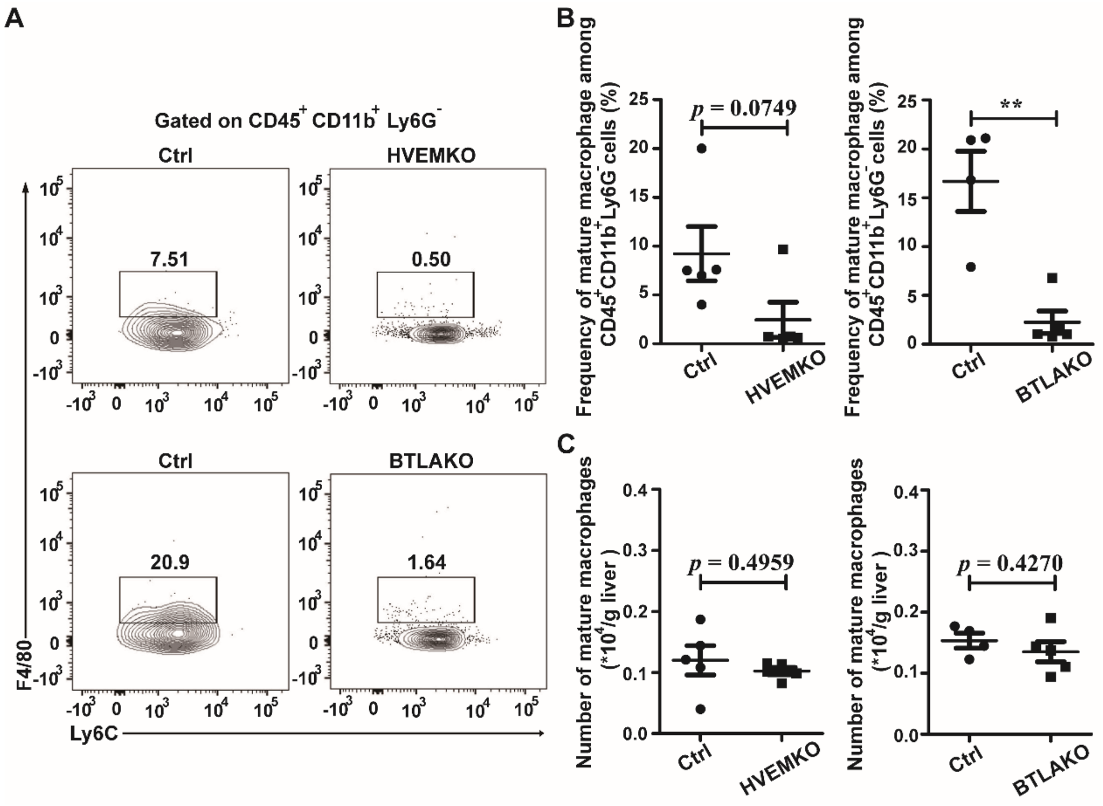


**Supplementary Figure S5. The percentage and number of mature macrophages in livers after 21 days of DDC-feeding.** Representative flow cytometry analysis (A), percentage (B), and cell number (C) of mature recruited macrophages ($CD45^{+}CD11b^{+}Ly6G^{-}F4/80^{+}Ly6C^{hi\text{-}lo}$) in livers from WT controls (ctrl), *HVEM*$^{-/-}$, and *BTLA*$^{-/-}$ mice after 21 days of DDC-feeding. n = 4-5 per group. ** $p < 0.01$.

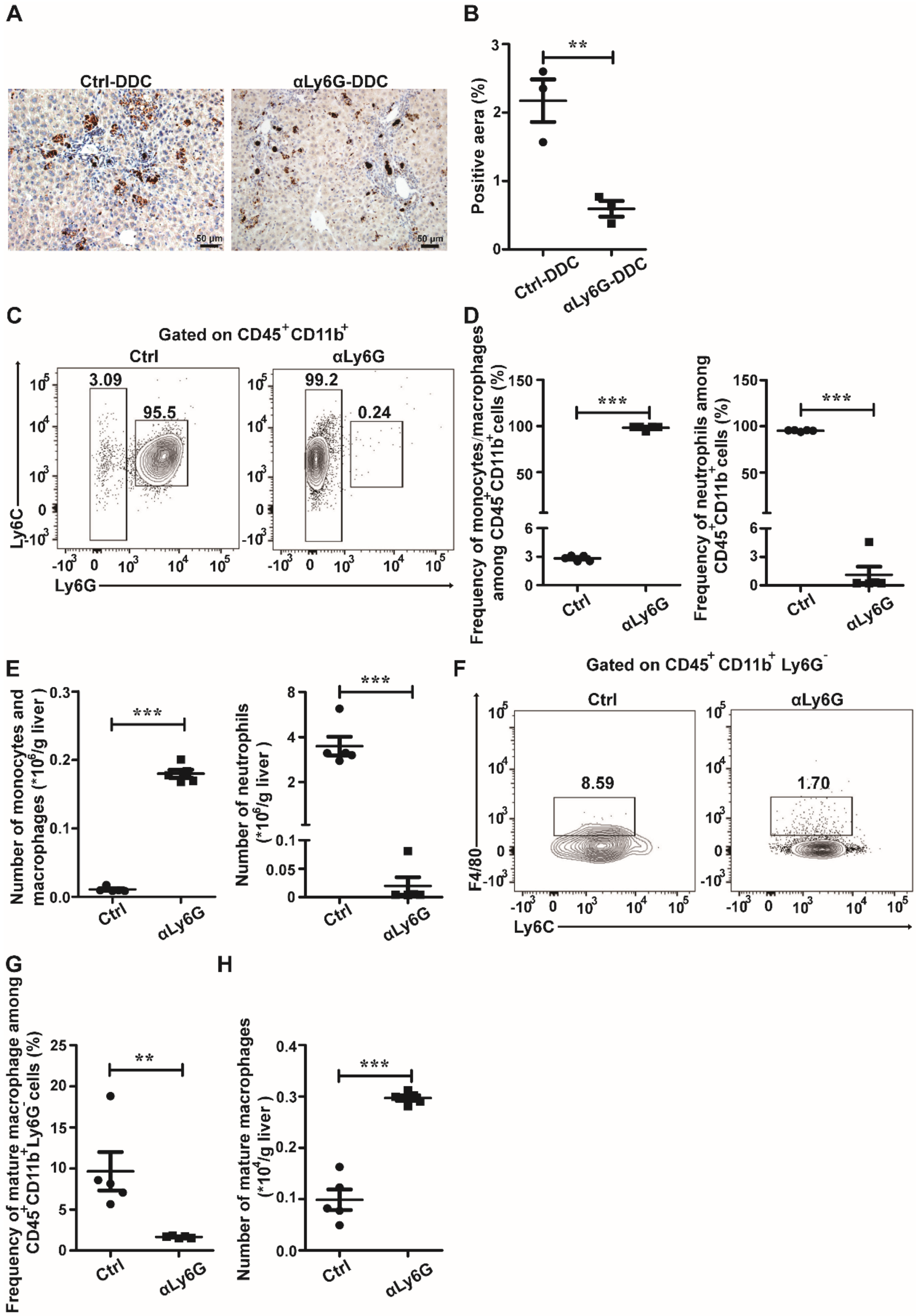


**Supplementary Figure S6. The neutrophil depletion efficacy of the anti-Ly6G antibody. (A)** Immunohistochemistry staining of MPO in liver sections from WT mice that were treated with a

control antibody (ctrl) or the anti-Ly6G antibody during DDC-feeding. n =3, scale bar = 50 µm. **(B)** The percentage of MPO-stained positive area (brown color) per visual field was calculated accordingly. n = 3. **(C-E)** Representative FACS contour plot (C) and graph of percentage (D) and absolute number (E) of neutrophils ($CD45^+CD11b^+Ly6G^+$) and monocyte/macrophage subset ($CD45^+CD11b^+Ly6G^-Ly6C^{hi-lo}$) in livers with or without anti-Ly6G antibody treatment during DDC-feeding were determined. n = 4 per group. **(F-H)** Representative FACS contour plot (F) and graph of percentage (G) and absolute number (H) of mature recruited macrophages ($CD45^+CD11b^+Ly6G^-F4/80^+Ly6C^{hi-lo}$) in livers with or without anti-Ly6G antibody treatment during DDC-feeding were tested. n = 4 per group. ** $p < 0.01$; *** $p < 0.001$.

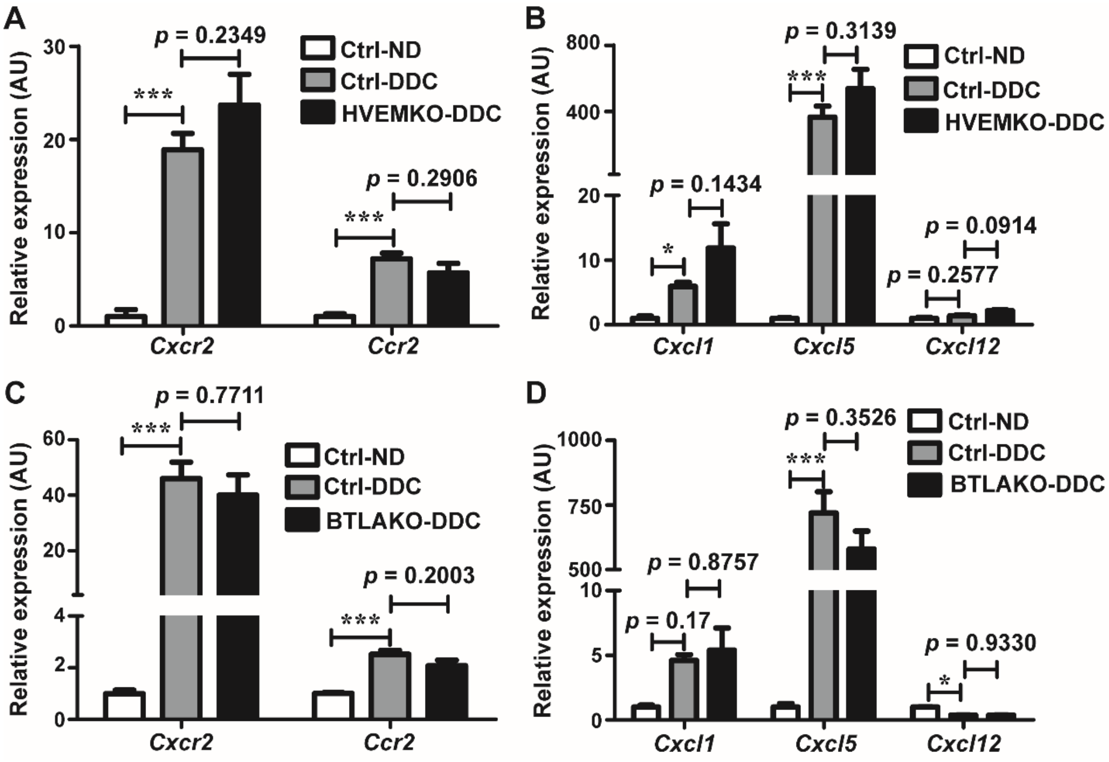


**Supplementary Figure S7. The relative mRNA levels of several major genes related to neutrophil chemotaxis are not influenced by the HVEM-BTLA axis during DDC-feeding. (A-D)** The relative mRNA expression levels of *Cxcr2*, *Ccr2*, *Cxcl1*, *Cxcl5,* and *Cxcl12* in the liver of WT controls (ctrl), *HVEM*$^{-/-}$, or *BTLA*$^{-/-}$ mice 21 days after DDC-feeding. ND, normal chow diet. n = 3-5 per group. Data were represented as the mean ± SEM. * $p < 0.05$; ** $p < 0.01$; *** $p < 0.001$.

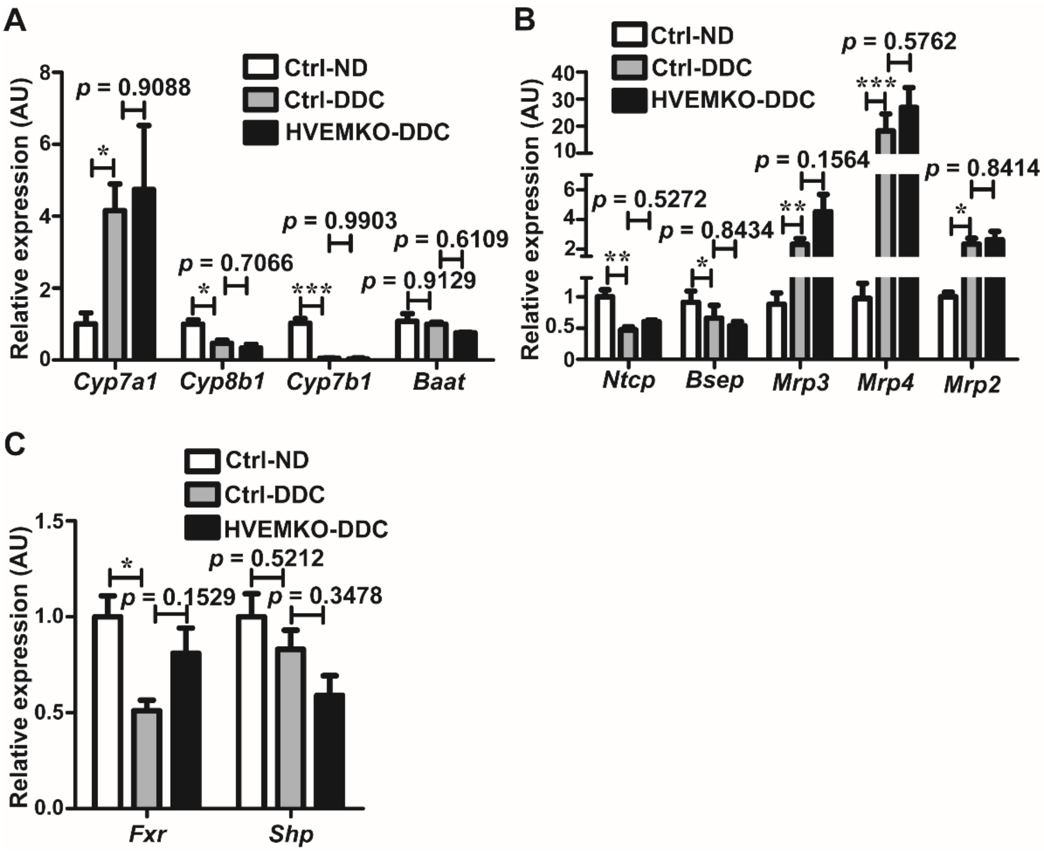


**Supplementary Figure S8. HVEM deficiency does not influence the mRNA expression levels of several essential genes related to bile acids synthesis, transport, and signaling during DDC-feeding. (A-C)** Real-time qPCR analysis of the relative hepatic mRNA expression levels of the indicated genes. Ctrl, WT control mice. ND, normal chow diet. n = 3-5. Data were represented as the mean ± SEM. * $p < 0.05$; ** $p < 0.01$; *** $p < 0.001$.

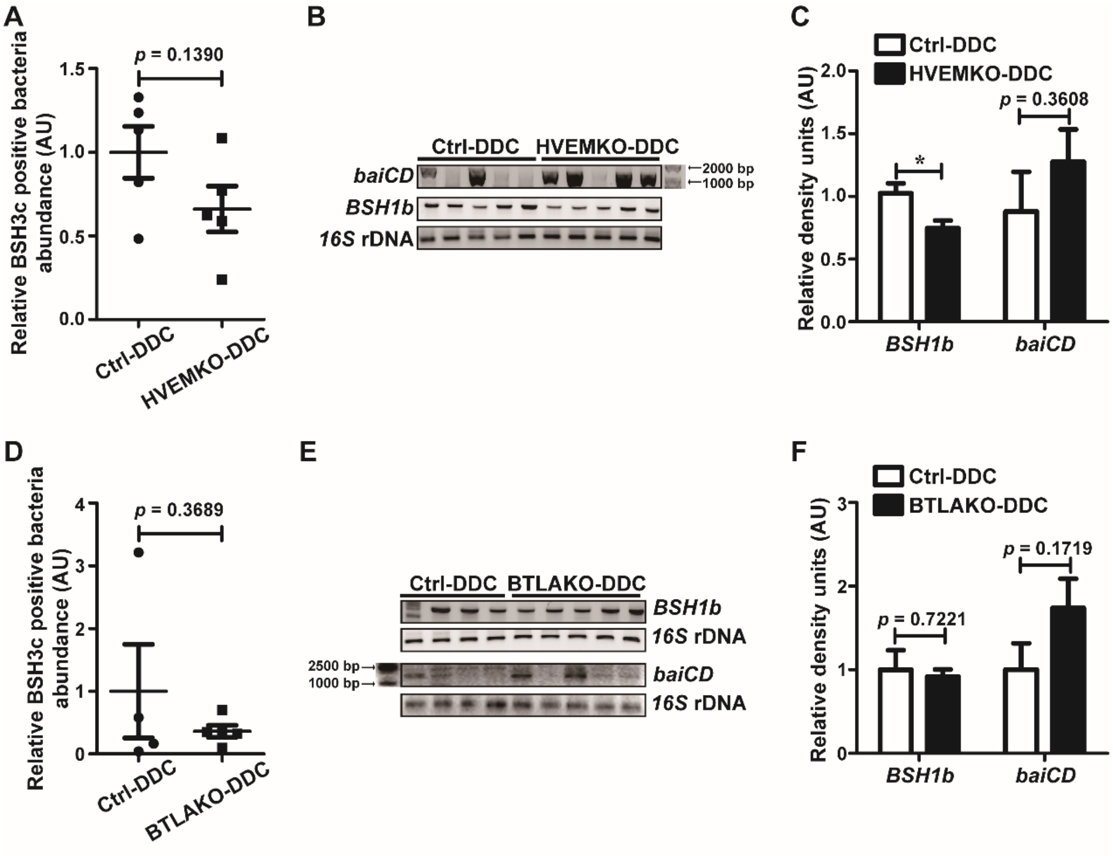


**Supplementary Figure S9. Relative abundance of bile acids-metabolizing microorganisms after DDC-feeding.** *HVEM*$^{-/-}$, *BTLA*$^{-/-}$, and their control mice (Ctrl) were fed with a normal chow diet (ND) or a DDC-containing diet for 21 days. n = 4-5. **(A, and D)** Relative abundance of the BSH group 3C bacteria in the cecal contents of *HVEM*$^{-/-}$ (A) and *BTLA*$^{-/-}$ (D) mice after 21 days of DDC-feeding, determined by real-time qPCR. **(B, C, E, and F)** Relative abundance of the BSH group 1B bacteria and 7-α-dehydroxylase-positive (encoding by *bai*CD operon) bacteria in the cecal contents of *HVEM*$^{-/-}$ (B and C) and *BTLA*$^{-/-}$ (E and F) mice after 21 days of DDC-feeding, determined by semi-qPCR. * $p < 0.05$; BSH, bile salt hydrolases.

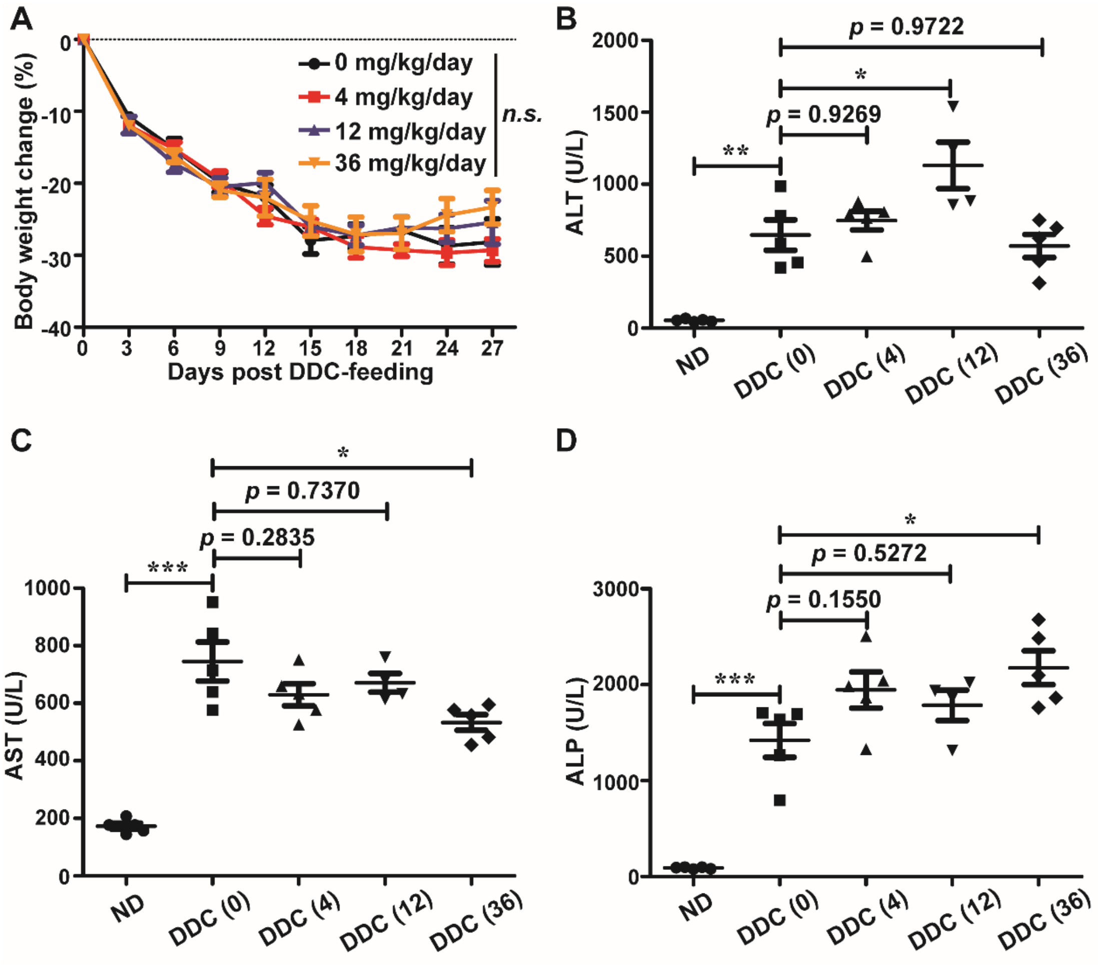


**Supplementary Figure S10. DCA shows no protection against DDC-induced cholestatic liver injury. (A)** Daily body weight changes of WT mice that were treated with DCA at the indicated dose during DDC-feeding. **(B-D)** Serum ALT (B), AST (C), and ALP (D) level 21 days after DDC-feeding. ND, normal chow diet; DDC(0), mice were fed with DDC and treated with 0 mg/kg/day DCA; DDC(4), mice were fed with DDC and treated with 4 mg/kg/day DCA; DDC(12), mice were fed with DDC and treated with 12 mg/kg/day DCA; DDC(36), mice were fed with DDC and treated with 36 mg/kg/day DCA. n = 4-5 per group. Data were represented as the mean ± SEM. * $p < 0.05$; ** $p < 0.01$; *** $p < 0.001$; ns, no statistical significance.

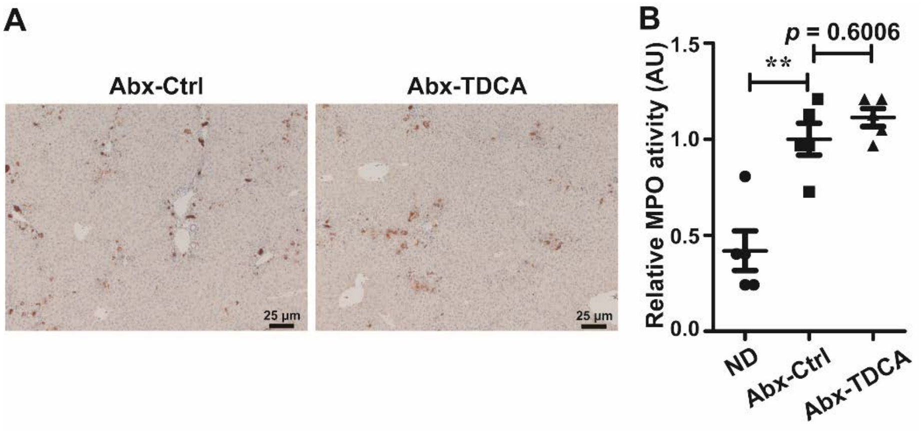


**Supplementary Figure S11. TDCA requires further biotransformation by the gut microbiota to increase intrahepatic neutrophils. (A-B)** Mice were fed with DDC in the diet and a cocktail of broad-spectrum antibiotics (Abx) in drinking water for 21 days. (A) Immunohistochemistry staining of MPO in liver sections from WT mice that were treated with vehicle (ctrl) or TDCA for 21 days during DDC-feeding. n = 5, scale bar = 25 µm. **(B)** MPO activity tests for liver tissues from the indicated mice. ND, normal chow diet. n = 5 per group. Data were represented as the mean ± SEM. ** $p < 0.01$.

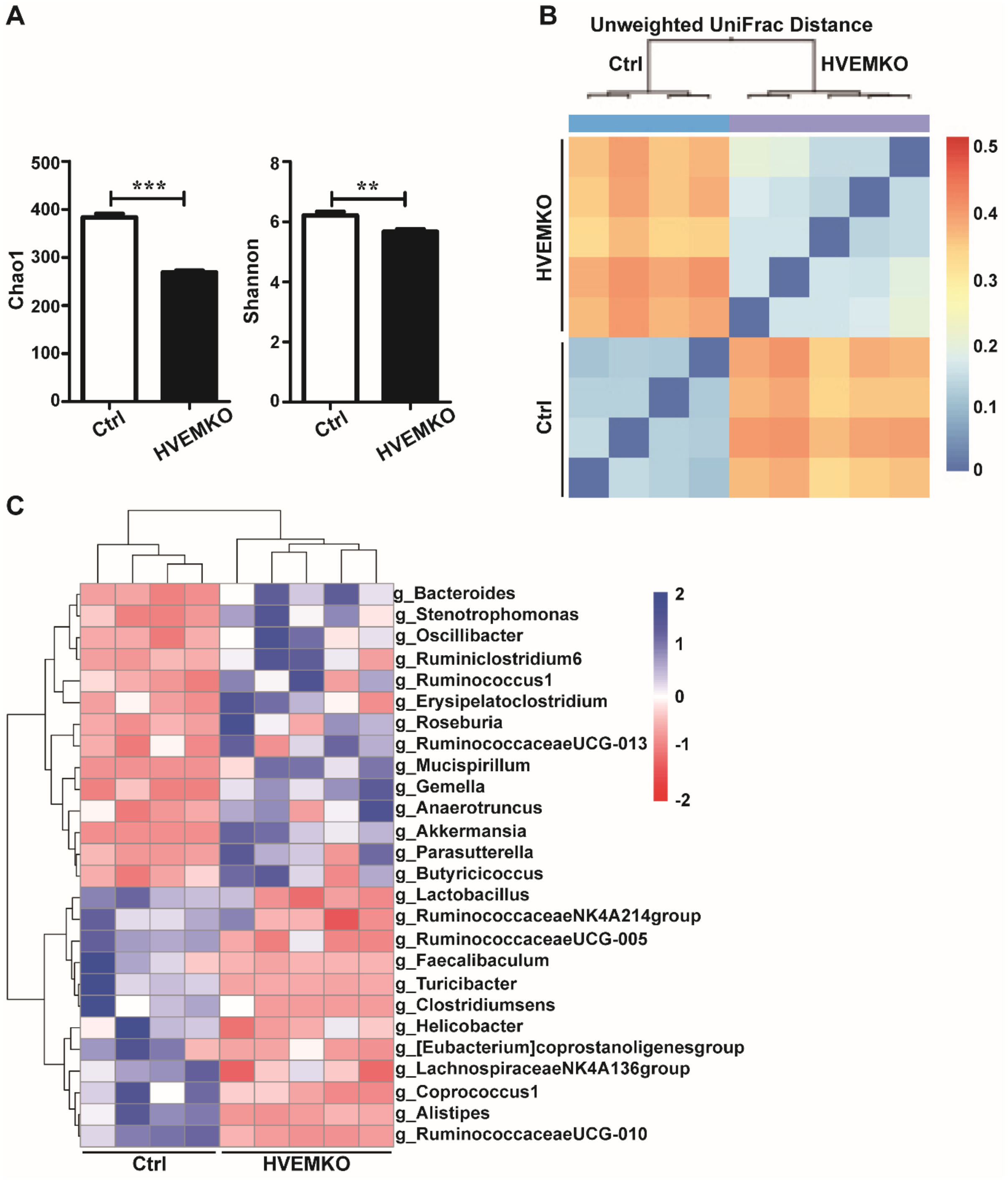


**Supplementary Figure S12. Uninfected *HVEM*$^{-/-}$ mice have reduced intestinal microbiota diversity.** The gut microbiota diversity was analyzed according to cecal content bacterial 16S rRNA gene sequencing data. **(A)** α-diversity is indicated by Chao1 (species richness) and Shannon index (species evenness). **(B)** Unweighted UniFrac distance indicates the microbiota landscape difference

between uninfected *HVEM*$^{-/-}$ and control (ctrl) mice. n = 4-5. ** $p < 0.01$; *** $p < 0.001$. **(C)** Differentially abundant taxa (at genus level) in naïve *HVEM*$^{-/-}$ and WT control mice.

## 1.2. Supplementary Table 1. List of real-time PCR primers used

| Genes | Primer sequence (5'-3') |
|---|---|
| *Light* | F: 5' ATCCCGCTACCCGAAGGAGTTA 3' |
| | R: 5' ACCAGGCGGTTTCCAGGCA 3' |
| *Hvem* | F: 5' CCTAACAGGGACCTTCTCACTTGG 3' |
| | R: 5' GCTATCCCAACTCCCACTATCACAA 3' |
| *Btla* | F: 5' AAGAGCCGACCCACATTTCCCT 3' |
| | R: 5' CATGAATGCCATTCGCACCG 3' |
| *β-Actin* | F: 5' TGAGAGGGAAATCGTGCGTGAC 3' |
| | F: 5' GCTCGTTGCCAATAGTGATGACC 3' |
| *Bacterial 16s* rDNA | F: 5' CGGTGAATACGTTCCCGG 3' |
| | R: 5' TACGGCTACCTTGTTACGACTT 3' |
| *Bsh1a* | F: 5' CACATATTGTGGCACGAACAATHGARTGGGG 3' |
| | R: 5' CTGTGCCCGGATACAGATTAACRTARTTRTT 3' |
| *Bsh3c* | F: 5' TTTTGGCCGAACACTGGAYTAYGARTT 3' |
| | R: 5' TCAACGGAGCCCAGAATATGRAARA AYTG 3' |
| *BaiCD* | F: 5' GGWTTCAGCCCRCAGATGTTCTTTG 3' |
| | R: 5' GAATTCCGGGTTCATGAACATTCTKCKAAG 3' |
| *Ccr2* | F: 5' TCTTCCTGCTCACATTACCATTC 3' |
| | R: 5' ATTGTCAGGAGGATAATGAAAAAGA 3' |
| *Cxcr2* | F: 5' CAAGTACACTGATCCAGAAGAGACA 3' |
| | R: 5' AAAGTCTGAGGCAGGATACGC 3' |
| *Cxcl1* | F: 5' CACCCAAACCGAAGTCATAGC 3' |
| | R: 5' GAAGCCAGCGTTCACCAGA 3' |
| *Cxcl5* | F: 5' TGGCATTTCTGTTGCTGTTC 3' |
| | R: 5' TGACTTCCACCGTAGGGC 3' |
| | F: 5' CACATCGCCAGAGCCAACG 3' |

| | |
|---|---|
| *Cxcl12* | R: 5' CTTTAATTTCGGGTCAATGCACAC 3' |
| *Asbt* | F: 5' CCGAGGATCTCAACCTGGTG 3' |
| | R: 5' GGTTGAAATCCCTTGTTTGTCTC 3' |
| *Ost-α* | F: 5' TCAAGATAACGCTGAGCATAGTGG 3’ |
| | R: 5' AAGCACCTGGAACAGAGCAAAC 3' |
| *Ost-β* | F: 5' ATGCGGCTCCTTGGAATTATT 3' |
| | R:5' TGGTGTTTCTTTGTCTTGTGGC 3' |
| *Bsep* | F: 5' GCTGCCAAGGATGCTAATGC 3' |
| | R:5' TGGGTTTCCGTATGAGGGC 3' |
| *Mrp2* | F: 5'AAACCTGATTGTCTTCTGCTCGG 3' |
| | R: 5' TGTGATGTTGAGGGCGTTGG 3' |
| *Mrp3* | F: 5' CAGCCTAAACATTCAAATCCCG 3' |
| | R: 5' CTTTACAGACACCACACCTTCCAG 3' |
| *Mrp4* | F: 5' CGTCACGGGATGTCAAGCG 3' |
| | R: 5' CAGAACAAGGGACCCGAAGG 3' |
| *Ntcp* | F: 5' ACCTGTCTAACCTCTTCACCCTG 3' |
| | R: 5' CTCCGTCGTAGATTCCTTTGC 3' |
| *Fxr* | F: 5' GCTTGATGTGCTACAAAAGCTG 3' |
| | R: 5' CGTGGTGATGGTTGAATGTCC 3' |
| *Shp* | F: 5' CTCATGGCCTCTACCCTCAA 3' |
| | R: 5' GGTCACCTCAGCAAAAGCAT 3' |
| *Cyp7a1* | F: 5' TTGTTCAAGACCGCACATAAAGC 3' |
| | R: 5' TCATCAAAGGTGGAGAGTGTATCGT 3' |
| *Cyp8b1* | F: 5' CCACCTGTTTCTGGGTCCTC 3' |
| | R: 5' GACTCTCCTCCATCACGCTGT 3' |
| *Cyp7b1* | F: 5' CTATGGAAGCCCTGCGTGAC 3' |
| | R: 5' CTTCTCGGATGATGCTGGAGTAT 3' |
| *Baat* | F: 5' GCTCTGGCTTACTGGAACTATGA 3' |
| | R: 5' TACAGTGGCTCTTATTTGTTTTAGG 3' |

| | |
|---|---|
| *Il17a* | F: 5' TGTCTGCCCTCCACAATGAAA 3' |
| | R: 5' AAGTCCACAGAAAAACAAACACGA 3' |
| *Il23* | F:5' GGTGGCTCAGGGAAATGTG 3' |
| | R: 5' GACAGAGCAGGCAGGTACAGA 3' |
